# Achieving 87% of Theoretical Output Charge Density by Optimizing Charge Behaviors in Polydimethylsiloxane/CaCu_3_Ti_4_O_12_-Based Triboelectric Nanogenerators

**DOI:** 10.34133/research.0921

**Published:** 2025-10-09

**Authors:** Jinyang Liu, Zhongkun Wang, Shuo Wang, Yuanzheng Zhang, Weikun Li, Song Zhao, Dongyang Li, Yonghui Wu, Hengyu Guo, Haiwu Zheng

**Affiliations:** ^1^ Henan Key Laboratory of High Efficiency Energy Conversion Science and Technology, Henan Province Engineering Research Center of Smart Micro-nano Sensing Technology and Application, School of Physics and Electronics, Henan University, Kaifeng 475004, China.; ^2^ School of Physics, Chongqing University, Chongqing 400044, China.

## Abstract

Triboelectric charge behaviors, including the generation, storage, and dissipation, play a pivotal role in determining the output charge density of triboelectric nanogenerators (TENGs). While numerous factors influence these behaviors, prior research has predominantly focused on individual parameters, often overlooking the interplay of multiple factors and their collective impact on output charge density. This study elucidates the underlying mechanism through which trade-offs among charge behaviors influence TENG performance by systematically investigating key parameters, including trap state density, relative dielectric constant, leakage current density, dielectric loss, and the effective work function of the tribo-dielectric layer. To optimize TENG performance, a polydimethylsiloxane/CaCu_3_Ti_4_O_12_ (PDMS/CCTO) composite membrane was employed as the tribo-dielectric layer, integrated with an external charge excitation strategy. The resulting external charge excitation TENG (ECE-TENG) achieved an output charge density reaching 87% of the theoretical maximum, surpassing previously reported values in the literature. Furthermore, the optimized ECE-TENG demonstrated practical utility by sustainably powering an electrochromic membrane for indoor light and temperature regulation. This work provides a comprehensive analysis of the synergistic effects arising from trade-offs among multiple factors, offering valuable insights for enhancing TENG output performance. The findings present a promising framework for future optimization strategies in triboelectric energy harvesting.

## Introduction

In recent years, the Internet of Things (IoTs), composed of ubiquitous sensor nodes, has undergone thriving development [1]. However, the traditional centralized, fixed, and orderly power supply mode based on the existing power grid is incompatible with the current development situation of IoTs due to its inflexible layout [2]. The widely distributed high-entropy mechanical energy in the environment is a promising candidate for addressing the power supply of sensor nodes [3]. Among various energy conversion technologies, triboelectric nanogenerators (TENGs) have garnered widespread application for harvesting high-entropy mechanical energy from the ambient environment with the advantages of low cost [4], abundant choice of materials [5], and excellent low-frequency output performance [6], thereby enabling the realization of powering IoT nodes [7]. Unfortunately, the output performance to be improved has become a serious obstacle to the utilization of TENGs as a standalone power source, as well as for large-scale commercial deployments [8].

The studies have revealed that output charge density (*σ*_out_) plays a pivotal role in boosting the output voltage and current, which is a key parameter for determining the TENG output [9,10]. In general, the molecular structure, surface morphology, dielectric performance, and trap state density of tribo-dielectric materials critically affect charge generation, storage, and dissipation behaviors, which essentially determine the *σ*_out_ of TENGs [11]. However, optimizing the TENG output through a single strategy usually leads to losing sight of the bigger picture [12–14]. Therefore, it is crucial to explore the potential mechanism of multifactor trade-offs on *σ*_out_ for further fabrication of high-performance TENGs.

Previous works have established that the *σ*_out_ of TENGs is constrained by 4 critical factors: contact efficiency, contact electrification ability, air breakdown threshold, and dielectric breakdown threshold [15]. Meanwhile, researchers have made many efforts to ameliorate the aforementioned limiting factors [16–18]. Among them, the external charge excitation TENG (ECE-TENG) gets rid of the limitation of the contact electrification effect and reaches a new milestone in output performance under high charge density [15]. For example, Hu and colleagues [19] improved the contact efficiency of ECE-TENG using homemade carbon silica gel electrodes, thereby further enhancing its *σ*_out_. Based on optimizing contact efficiency, Wang and colleagues [20] introduced a high dielectric constant poly(vinylidene fluoride-trifluoroethylene-chlorofluoroethylene) [P (VDF-TrFE-CFE)] as a triboelectric layer, which significantly improved both charge generation and storage capabilities. However, it was found that the measured *σ*_out_ of ECE-TENG was much lower than the theoretical value. Afterward, Hu and colleagues [21] successfully suppressed the effect of deposited charges on *σ*_out_ by implementing the charge capture failure strategy. Wang et al. [22] pointed out that by effectively lowering leakage current density, the charge dissipation can be significantly reduced, thereby increasing the measured *σ*_out_ to 74% of the theoretical value. Nevertheless, this approach was also found to result in a reduction in total charge storage capacity. Although extensive research has improved the TENG output through the optimization of charge excitation strategies, the effect mechanisms of the trade-offs among multiple factors such as trap state density, relative dielectric constant, leakage current density, dielectric loss, and the work function difference on *σ*_out_ remain to be thoroughly investigated.

In this work, the synergistic mechanism by which the *σ*_out_ of TENG is modulated by a combination of charge generation, storage, and dissipation factors has been revealed. The high dielectric organic–inorganic composite was employed as the tribo-dielectric layer of the contact-separated TENG (CS-TENG) to enhance the charge storage capacity, thereby increasing *σ*_out_. In spite of this, the maximum *σ*_out_ was merely 17% of the theoretical value. Experimental results indicate that this discrepancy is intimately linked to the inadequate contact electrification ability of composite membranes. Next, an ECE-TENG with a function of charge accumulation was fabricated, aiming to be free from the limitation of the contact electrification effect. However, inappropriate contents of inorganic fillers inside the composite membrane will lead to increased trap state density, leakage current density, and dielectric loss in the composite membrane, and these factors reduce the *σ*_out_ of ECE-TENG and adversely affect its output performance. After comprehensive consideration of multiple factors, the optimal ECE-TENG exhibited the highest *σ*_out_ of 236 μC m^−2^, amounting to 87% of the theoretical maximum. The indoor light-temperature regulation system (ILTRS) constructed from the optimized ECE-TENG and electrochromic membranes can freely switch between transparent and opaque states. The opacity and transmittance of electrochromic membrane powered by ECE-TENG are higher than 62% and 87% at wavelengths of 405, 523, and 635 nm, respectively, demonstrating the outstanding ability of the ILTRS to regulate light intensity. After 1,200 s of illumination, the temperature inside the building model is 16 °C lower than the outside. This research reveals the collaborative effects of multiple factors on the TENG output and provides a feasible strategy for developing high-performance TENG.

## Results and Discussion

### Research overview

**Fig. 1.**
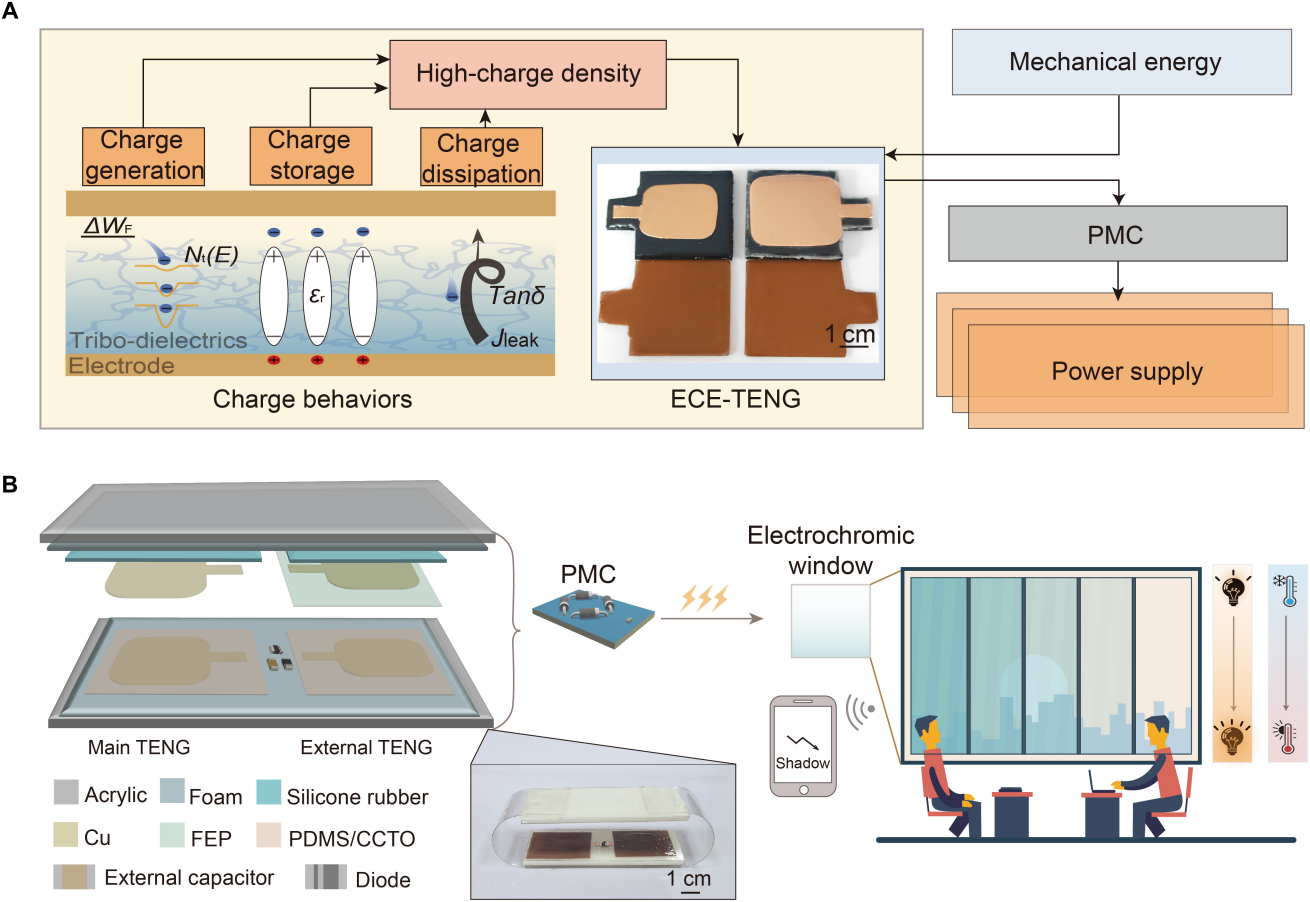
Schematic illustration of high-performance ECE-TENG and demonstration of ILTRS. (A) Three paths for achieving high output charge density (*σ*_out_), along with the workflow of ECE-TENG in harvesting mechanical energy to power electronic devices. (B) Application scenario of the ILTRS for indoor light-temperature regulation.

As sketched in Fig. 1A, previous studies have demonstrated that key parameters of composite membranes—such as those related to charge generation {trap state density [*N*_t_(*E*)] and the work function difference (*ΔW*_F_) between tribo-dielectric layers}, charge storage [relative dielectric constant (*ε*_r_)], and charge dissipation [leakage current density (*J*_leak_) and dielectric loss (*tanδ*)]—all exert significant influence on the output performance of TENG [11–14,19–22]. To regulate the multi-parameter coupling of charge behavior and achieve optimal output performance of TENG, the polydimethylsiloxane/CaCu₃Ti₄O₁₂ (PDMS/CCTO) composite membranes prepared by spin-coating in Fig. S1 served as the tribo-dielectric layer of ECE-TENG, and the fabrication method is outlined in Materials and Methods. Specifically, ECE-TENG consists of a main TENG, an excitation TENG, and a charge excitation circuit, as depicted in the schematic diagram in Fig. 1B. Here, Cu serves as both the electrode and tribo-dielectric layer of the main TENG, while PDMS/CCTO functions as the other tribo-dielectric layer. The PDMS/CCTO and commercial fluorinated ethylene propylene (FEP) membrane are employed as the tribo-dielectric layer of the external TENG. Afterward, an ILTRS comprising an ECE-TENG, an electrochromic membrane, and a power management circuit (PMC) was constructed. With the assistance of PMC, ECE-TENG provides continuous and stable power to the electrochromic membrane, enabling it to switch between transparent and opaque states to address the varying requirements for light intensity and temperature across different conditions. Simultaneously, a brightness sensor wirelessly transmits indoor light intensity data to a terminal for real-time monitoring.

### Characterization of PDMS/CCTO composite membranes

**Fig. 2.**
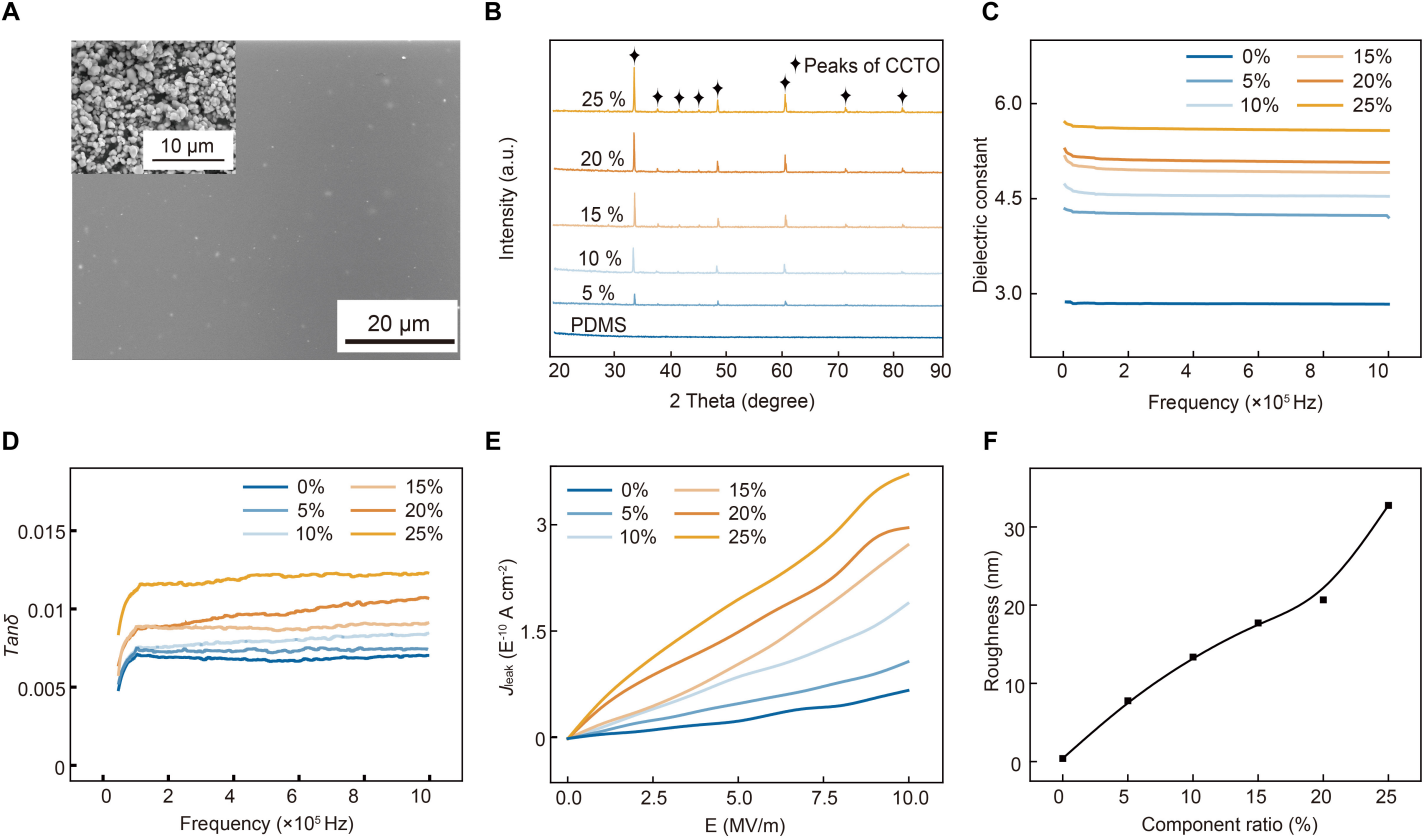
Characterization of PDMS/CCTO composite membranes. (A) SEM images of the PDMS/CCTO-15 wt% composite membrane. The inset shows an SEM image of the CCTO particles. (B) XRD patterns, (C) *ε*_r_, (D) *tanδ*, (E) *J*_leak_, and (F) surface roughness of PDMS/CCTO composite membranes with different CCTO contents.

To investigate the effect of inorganic filler on the dielectric performances of organic–inorganic composite membranes, PDMS/CCTO membranes with different contents of CCTO particles were prepared using a spin-coating method as outlined in Materials and Methods. Figure 2A shows the scanning electron microscopy (SEM) image of the PDMS/CCTO-15 wt% composite membrane, indicating that CCTO particles have good compatibility with the PDMS matrix. The inset in Fig. 2A provides the SEM image of the CCTO particles, and the particle size distribution analysis in Fig. S2A reveals an average particle size of approximately 1.17 μm. Furthermore, energy-dispersive x-ray spectroscopy (EDS) was used to analyze the elemental distribution of the CCTO particles in Fig. S2B. The uniform distribution of Ti, O, Cu, and Ca elements confirms the high quality of the CCTO. Figure 2B presents the x-ray diffraction (XRD) patterns of the organic-inorganic composite membranes with different CCTO contents. The intensity of the characteristic peaks of CCTO increases with the increase of CCTO contents, confirming the successful incorporation of CCTO particles into the PDMS matrix [23]. The dielectric spectrum of the PDMS/CCTO composite membranes with different CCTO contents is displayed in Fig. 2C. The *ε*_r_ of the composite membranes is significantly enhanced compared to pure PDMS, increasing from 2.7 to 5.18 at low frequency (100 Hz) as the CCTO contents increase, which is ascribed to the interfacial polarization induced by the incorporation of CCTO into the PDMS matrix [15,24]. However, as shown in Fig. 2D, the increase in CCTO contents also leads to higher *tanδ*, resulting from relaxation polarization and conductive losses [25]. Additionally, Fig. 2E demonstrates that the *J*_leak_ of the composite membranes increases with the increase of CCTO contents, which may be due to particle agglomeration and introduced defects [26]. As a result, on the one hand, the PDMS/CCTO composite membranes exhibit improved *ε*_r_, which is favorable for improving the TENG output [27]. In addition, this enhancement is accompanied by an increase in its *tanδ* and *J*_leak_, and both parameters are not favorable to the TENG output [28]. Surface roughness is another critical factor affecting the TENG output. As shown in Fig. 2F and Fig. S3, the surface roughness of the PDMS/CCTO composite membranes increases with higher CCTO contents, which is beneficial for enhancing the effective contact area and thereby improving the TENG output [29]. To further investigate the effect of surface roughness on the electrical output performance of TENG, a PDMS/CCTO-15 wt% composite membrane with a microstructured surface was prepared using a corresponding template, yielding an average surface roughness (Ra) of 41.489 nm in Fig. S3B. As illustrated in Fig. S3C, the output charge density (*σ*_out_) of the microstructured composite membrane increased by approximately 30% compared to that of the flat membrane, demonstrating that enhanced surface roughness of the friction layer positively influences the output performance of TENG.

### Output performance of CS-TENG

**Fig. 3.**
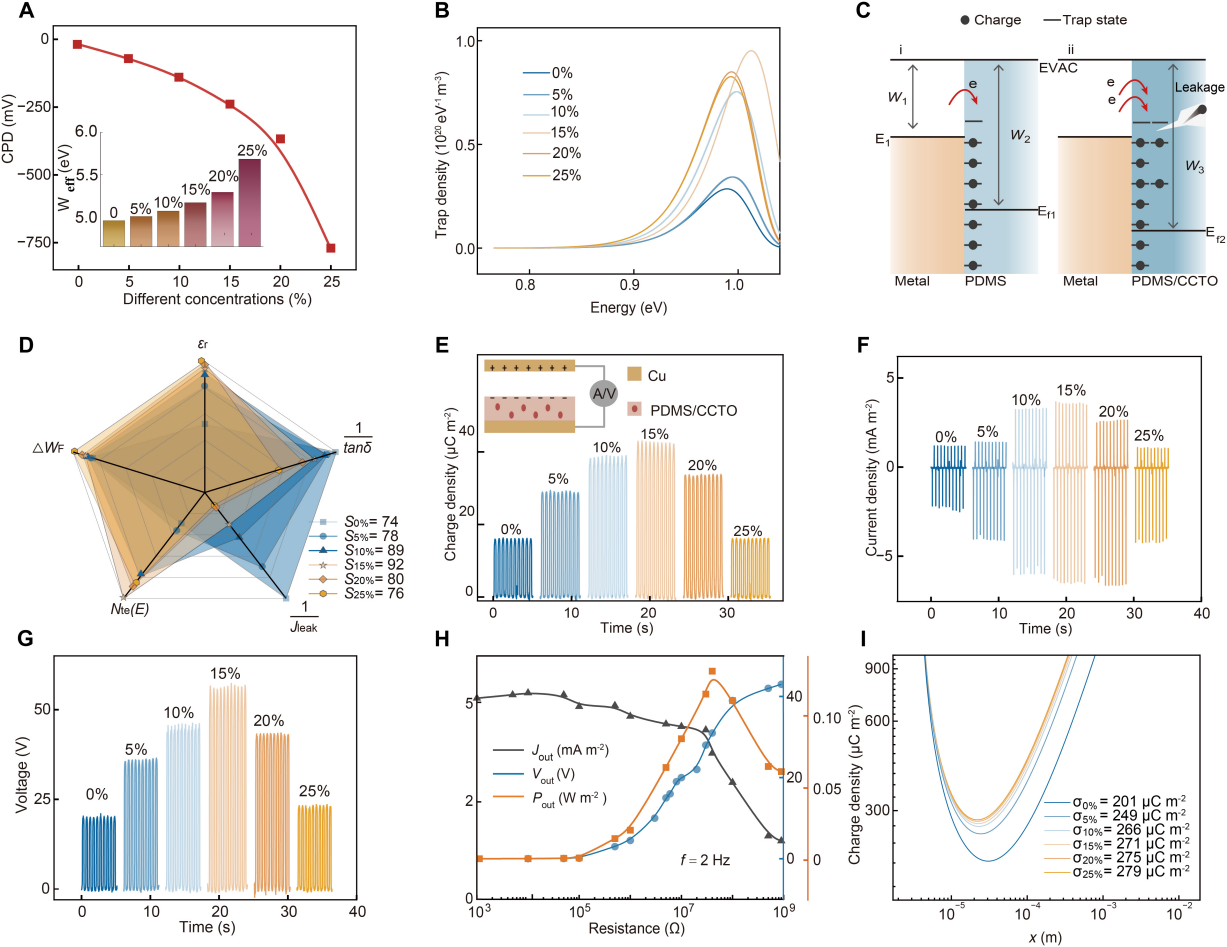
Electrical output characteristics of CS-TENG prepared from PDMS/CCTO composite membranes. (A) Contact potential difference (CPD) and corresponding effective work function (*W*_eff_) of tribo-dielectric layers. (B) Deep trap state energy distribution and density distribution for electrons with different CCTO contents. (C) Energy band diagrams at the interface between the Cu and PDMS/CCTO layers. (D) Radar chart of multiple factors of composite membranes with different CCTO contents. (E) Output charge density (*σ*_out_), (F) short-circuit current density (*J*_SC_), and (G) open-circuit voltage (*V*_OC_) of ECE-TENG with different CCTO contents. (H) Dependence of the output performance of CS-TENG with PDMS/CCTO-15 wt% on the external variable resistance. (I) Theoretical charge densities of tribo-dielectric layers with different CCTO contents.

The effects of inorganic filler on *W*_eff_ and *N*_t_(*E*) have also been analyzed. The contact potential difference (CPD) of PDMS/CCTO composite membranes with different CCTO contents and the corresponding *W*_eff_ are recorded in Fig. 3A. Detailed kelvin probe force microscopy (KPFM) results and the method for calculating *W*_eff_ are presented in Fig. S4 and Note S1. With the increase of CCTO contents, the CPD of the composite membrane gradually decreases, and *W*_eff_ gradually increases, which ultimately causes an increase in the work function difference (*ΔW*_F_) between PDMS/CCTO composite membranes and Cu electrode. According to previous literature reports, the larger the work function difference, the more charge transfer occurs during contact [30]. In addition, based on the isothermal surface potential decay theory, the relationships between the trap state energy distribution and the density distribution were calculated using the surface potential decay curves shown in Fig. S5A. The specific calculation method is detailed in Note S2. The results indicate that the electron shallow trap state density is much lower than that of the electron deep trap state density in Fig. 3B and Fig. S5B. Therefore, *N*_te_(*E*) is dominated by the electron deep trap state density [31]. From Fig. 3B, with the increase of CCTO contents, the *N*_te_(*E*) of PDMS/CCTO-15 wt% is the highest before declining, which is conducive to improving the charge transfer amount [32]. With the addition of CCTO particles, a large number of high potential barrier matrix/particle interfaces are generated in the PDMS/CCTO composite membrane, which can hinder electron movement and effectively increase *N*_te_(*E*). However, the agglomeration phenomenon caused by excessive CCTO particles can lead to an increase in the conductive path, resulting in a gradual decrease in *N*_te_(*E*) [26]. Figure 3C sketches the energy band diagrams at the interface between the Cu and PDMS/CCTO layers for analyzing the effect of *ΔW*_F_ and *N*_te_(*E*) on the charge transfer. The larger the *W*_eff_ and *N*_te_(*E*) of the composite membranes, the more electrons need to be transferred from the metal to the tribo-dielectric layer to bring the Fermi levels of the 2 materials into equilibrium [32]. As a result, more charge transfer occurs during metal contact in PDMS/CCTO composite membranes (Fig. 3 Ci) than in pure PDMS composite membranes (Fig. 3 Cii). Furthermore, as shown in Fig. 2C, the increase in the *ε*_r_ of high-content PDMS/CCTO composite membrane is related to their enhanced polarization ability, which not only improves capacitance but also promotes charge induction between the upper and lower electrodes, enhancing the CS-TENG output [33]. However, excessive CCTO particles can lead to increased *tanδ* and *J*_leak_, which intensifies internal charge dissipation in the tribo-dielectric layer and ultimately results in a decrease in the *σ*_out_ of CS-TENG in Fig. 2D and E [34]. Previous reports have indicated that *W*_eff_, *N*_t_(*E*), and *ε*_r_ favorably improve the CS-TENG output, while *tanδ* and *J*_leak_ deteriorate the output performance. To more visually assess the combined effect of the above multiple factors on the CS-TENG output, each factor was normalized and presented in the form of a radar chart as shown in Fig. 3D. In this case, the normalized values of *tanδ* and *J*_leak_, which are detrimental to the output performance, are shown in inverted form. The area of the closed graph consisting of the 5 factors for each component composite membrane is shown in Fig. 3D. The area of PDMS/CCTO-15 wt% composite membrane reaches the maximum value, and it can be reasonably hypothesized that CS-TENG prepared with PDMS/CCTO-15 wt% will exhibit optimal output performance.

To validate this hypothesis, the CS-TENG output was characterized in Fig. 3E, with its structure schematically shown in the insert. Figure S6 gives the optical images of the tribo-dielectric layers with different CCTO contents. The CS-TENG working principle is the coupling of contact electrification and electrostatic induction, as presented in Fig. S7 [35]. The effect of different CCTO contents inside composition membranes on TENG output was tested, as shown in Fig. 3E to G. The changing trends in *σ*_out_, *J*_SC_, and *V*_OC_ of CS-TENG are similar, showing a trend of first increasing and then decreasing with the increase of CCTO contents. When the CCTO content is 15 wt%, the samples exhibit optimal values of *σ*_out_, *J*_SC_, and *V*_OC_ of 45 μC m^−2^, 6 mA m^−2^, and 55 V, respectively. In this situation, the maximum instantaneous output power density (*P*_out_) is 0.15 W m^−2^, as presented in Fig. 3H. Notably, the CS-TENG output is consistent with the changing trend of the area of the radar chart in Fig. 3D, indicating that the CS-TENG output can be improved by increasing the values of *ΔW*_F_, *N*_te_(*E*), and *ε*_r_, and decreasing the values of *tanδ* and *J*_leak_. Briefly, the balance of the above parameters determines the TENG output.

According to Paschen’s law, the theoretical maximum charge density of CS-TENG increased gradually from 201 to 279 μC m^−2^ with the rise of the CCTO contents, as illustrated in Fig. 3I. The specific calculation process is recorded in Note S3 [36]. It can be seen that, theoretically, the charge storage capability of the PDMS/CCTO composite membrane can be improved by increasing *ε*_r_. Notably, although the output performances of the PDMS/CCTO-15 wt% composite membrane showed the optimal value among all the samples, as shown in Fig. 3E to G, *σ*_out_ is 45 μC m^−2^, which is only 17% of the theoretical value. Considering that the *V*_oc_ of PDMS/CCTO-15 wt% composite membrane is 55 V, the equivalent electric field experienced by its composite membrane is 0.55 MV/m. From the data in Fig. 2E, the *J*_leak_ of the PDMS/CCTO-15 wt% composite membrane under this electric field is negligible, indicating good charge storage capability. Therefore, the difference between the test and theoretical values of *σ*_out_ may not be related to *J*_leak_, but rather caused by the inability of the composite membrane to provide more charges due to insufficient contact electrification ability, which may be attributed to the non-uniformity and randomness of the contact between the upper and lower tribo-dielectric layers limiting the generation of friction charges [14]. To validate the effect of surface charge density on *σ*_out_, the charges were injected into the surface of the PDMS/ CCTO-15 wt% composite membrane by an ion gun, as shown in Fig. S8. In this case, the *σ*_out_ of CS-TENG gradually tends to saturation to 248 μC m^−2^ with increasing ion implantation cycles. *σ*_out_ after saturation reached 91% of the theoretical value, which was higher than 17% before ion implantation. Experimental results in Fig. S8 prove that the main reason for the difference between the test and the theoretical value is the insufficient contact electrification ability of the PDMS/CCTO composite membrane.

### Output performance of ECE-TENG

**Fig. 4.**
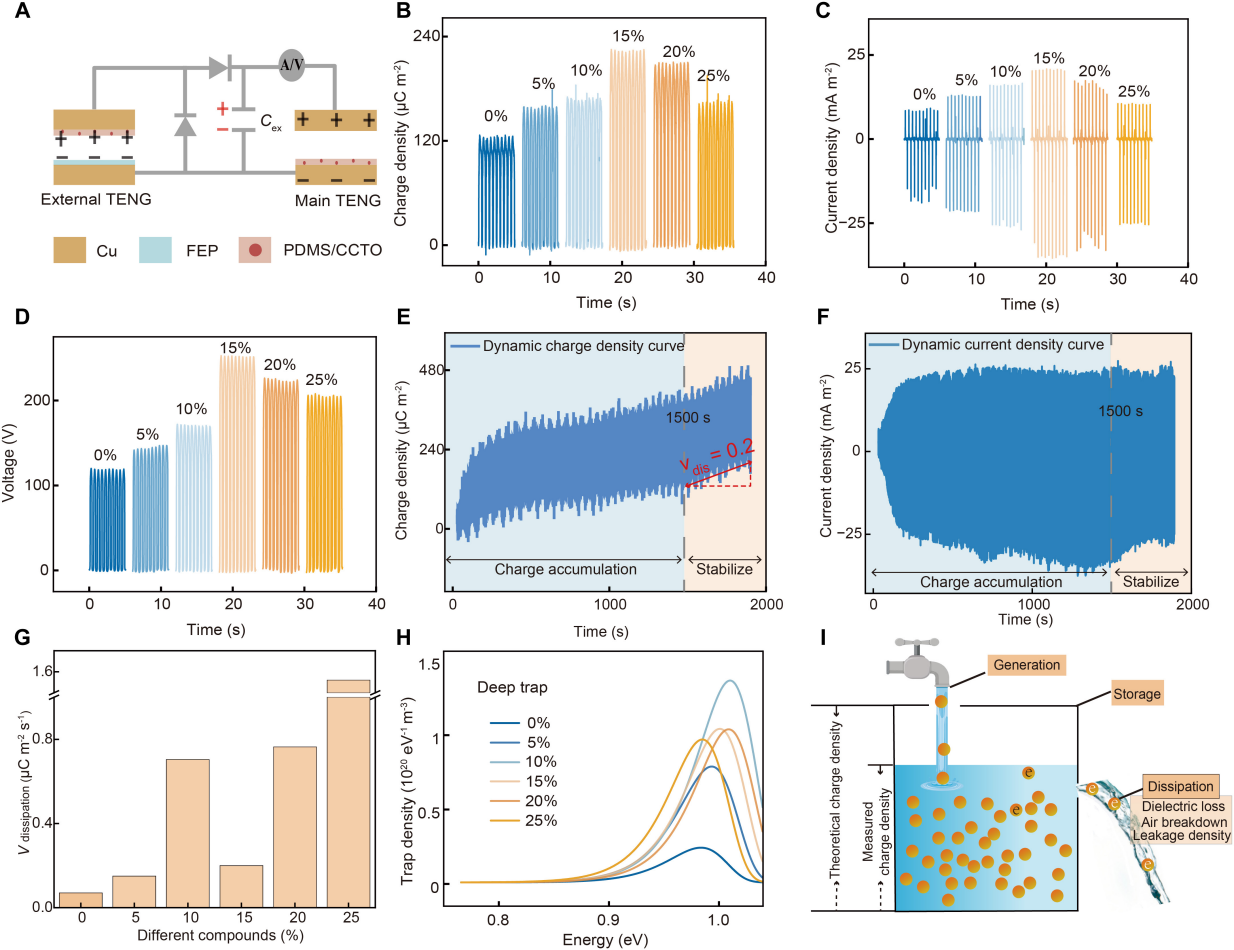
Output characteristics of ECE-TENG prepared by PDMS/CCTO composite membranes. (A) Structural diagram of ECE-TENG. (B) *σ*_out_, (C) *J*_SC_, and (D) *V*_OC_ of ECE-TENG with different CCTO contents. (E) Dynamic charge density and (F) dynamic current density of ECE-TENG with PDMS/CCTO-15 wt%. (G) Charge dissipation rate of ECE-TENG with different CCTO contents. (H) Deep trap state energy distribution and density distribution for holes with different CCTO contents. (I) Diagram for the comprehensive effects of multiple factors on the *σ*_out_ of ECE-TENG.

To get rid of the limitation of *σ*_out_ caused by contact electrification in the preceding analysis, a charge excitation strategy of ECE-TENG was proposed. As shown in Fig. 4A, ECE-TENG was prepared, which consisted of an external TENG, a main TENG, a half-wave rectifier bridge, and an external capacitor (*C*_ex_). Figure S9 shows the working principle of ECE-TENG [15]. The charges generated by the external TENG are stored in *C*_ex_ through a charge excitation circuit (Fig. S9A). During the contact process of ECE-TENG (Fig. S9B), the capacitance of both the main TENG and the external TENG gradually increases as the distance between the 2 tribo-dielectrics decreases. Due to the unidirectional conductivity of the diode, the charges stored in *C*_ex_ are transferred to the main TENG until it fully contacts (Fig. S9D). Then, during the separation process of ECE-TENG (Fig. S9C), the capacitance of the main TENG gradually decreases, and the charges flow back to *C*_ex_ until complete separation (Fig. S9A). Compared to CS-TENG, the external TENG continuously replenishes the charges to *C*_ex_, and the *σ*_out_ of the main TENG ultimately tends to a stable state due to the limitation of air breakdown [37]. To provide sufficient transfer charges and faster charge accumulation rates, a PDMS/CCTO-15 wt% composite membrane and FEP with stronger electronegativity were selected as the tribo-dielectric materials to improve the external TENG output. The *V*_OC_, *σ*_out,_ and *J*_SC_ of the external TENG are 150 V, 70 μC m^−2^, and 12 mA m^−2^, respectively, as presented in Fig. S10.

The effect of CCTO contents on the ECE-TENG output has been investigated in Fig. 4B to D. The *σ*_out_, *J*_SC_, and *V*_OC_ of ECE-TENG show that the highest values are 225 μC m^−2^, 35 mA m^−2^, and 254 V, respectively, for ECE-TENG with PDMS/CCTO-15 wt%. In comparison to CS-TENG, ECE-TENG exhibits a significant enhancement in output performance, demonstrating 5-, 5.8-, and 4.6-fold increases in *σ*_out_, *J*_SC_, and *V*_OC_, respectively. At the same time, the *σ*_out_ of ECE-TENG in Fig. 4B achieves 83% of the theoretical value calculated in Fig. 3I, which is much higher than 17% of CS-TENG in Fig. 3E to G. This enhancement can be attributed to the charges accumulated in *C*_ex_ being transferred directly to the electrodes of the main TENG, overcoming contact electrification limitations and maximizing *σ*_out_ [20]. But the output performance decreases at higher contents, which may be attributed to charge dissipation [12].

The dynamic charge density curve of the optimal ECE-TENG exhibits an increasing baseline, as shown in Fig. 4E [18]. This phenomenon can be ascribed to the surface deposition charge and internal charge loss of the tribo-dielectric layer, which results in a decrease in the charge transferred from the main TENG back to *C*_ex_ during the separation process [38], and ultimately adversely affects *σ*_out_. Figure S11 provides analyses of specific charge dissipation mechanisms. The charge dissipation rate (*v*_dis_) is defined as the amount of charge dissipated per unit of time for investigating the effect of different factors on the *σ*_out_ of ECE-TENG:

$$V_{\mathrm{dis}}=\frac{\Delta Q}{\Delta t}$$ (1)

where Δ*Q* is the amount of charge dissipated during the separation process and Δ*t* is the time interval. The dynamic current density curve of ECE-TENG with PDMS/CCTO-15 wt% in Fig. 4F tends to attenuate to equilibrium after 1,500 s, which is the same as the trend of dynamic charge density. It is reported that *v*_dis_ is determined by the hole trap state density [*N*_th_(*E*)] on the surface of the tribo-dielectric layer, as well as *J*_leak_ and *tanδ* inside the tribo-dielectric layer [23]. To verify the above analysis, combined with the charge dissipation curve presented in Fig. S12, the *v_dis_* of ECE-TENGs with different CCTO contents has been calculated in Fig. 4G. The *v_dis_* of the PDMS/CCTO-10 wt% composite membrane reaches its local maximum value of 0.70 μC m^−2^ s^−1^, which is attributed to the *N*_th_(*E*) of the composite membrane reaching a maximum value at this point in Fig. 4H and Fig. S13B. Moreover, with the increase of CCTO contents, the *J*_leak_ and *tanδ* of the PDMS/CCTO composite membranes also increase, resulting in the maximum *v_dis_* of 1.56 μC m^−2^ s^−1^ at PDMS/CCTO-25 wt%, as shown in Fig. 2D and E.

The above analysis indicates that the *σ*_out_ of ECE-TENG is not only positively affected by *ε*_r_ but also negatively affected by factors such as *N*_th_(*E*), *J*_leak_, and *tanδ*. In brief, the *σ*_out_ of ECE-TENG is affected by the trade-offs among charge generation, charge storage, and charge dissipation in Fig. 4I. In brief, ECE-TENG with PDMS/CCTO-15 wt% reaches the optimal balance among charge generation, storage, and dissipation, thus enabling its output performance to be superior to that of other ECE-TENGs with CCTO contents instead of 15 wt%.

**Fig. 5.**
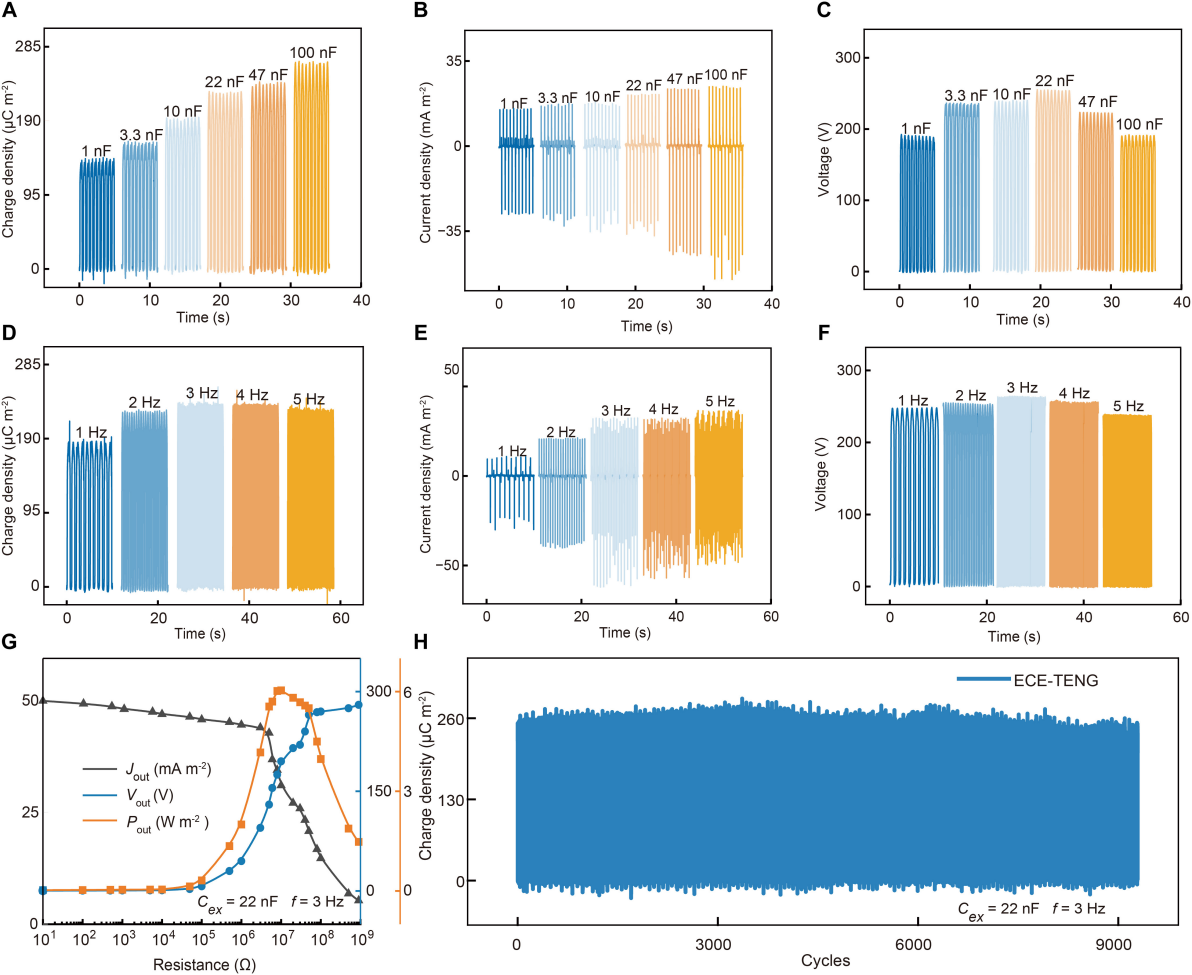
Optimizing the output performance of ECE-TENG. Dependence of *C_ex_* on the (A) *σ*_out_, (B) *J*_SC_, and (C) *V*_OC_ of ECE-TENG. Dependence of operating frequencies (*f*) on the (D) *σ*_out_, (E) *J*_SC_, and (F) *V*_OC_ of ECE-TENG. (G) Dependence of the output performance of ECE-TENG with PDMS/CCTO-15 wt% on the external variable resistance. (H) Durability test of ECE-TENG with PDMS/CCTO-15 wt%.

According to Note S3, *C*_ex_ affects the theoretical maximum charge density in the charge excitation circuit, serving as a key limiting factor for the ECE-TENG output. As depicted in Fig. 5A and B, the *σ*_out_ and *J*_SC_ of ECE-TENG increase with increasing *C*_ex_, which is due to the suppression of air breakdown by large *C*_ex_. Notably, the large capacitor also causes the charge excitation voltage to be suppressed, which causes the *V*_OC_ of ECE-TENG to reach a maximum value of 254 V at a *C*_ex_ of 22 nF instead of increasing sustainably as *C*_ex_ increases [39], as shown in Fig. 5C. The larger *C*_ex_ has a greater ability to store charge in Fig. S14, so it takes a longer time for ECE-TENG to reach the saturation value of *σ*_out_. Considering the effect of *V*_OC_ and the saturation time, a *C*_ex_ of 22 nF has been selected for subsequent testing. Furthermore, the ECE-TENG output is also affected by different operating frequencies (*f*), as shown in Fig. 5D to F. The *σ*_out_, *J*_SC_, and *V*_OC_ of ECE-TENG reach their optimal values of 236 μC m^−2^, 60 mA m^−2^, and 264 V at the *f* of 3 Hz, respectively. The reason is that too low and too high *f* can result in slow charge accumulation and short contact time, which affects the ECE-TENG output. Therefore, under optimized conditions where charge generation, storage, and dissipation reach equilibrium, and with suitable *C*_ex_ and *f* in the charge excitation circuit, the *σ*_out_ of ECE-TENG reaches 87% of the theoretical value, which is a significant improvement over the published literatures in Table S1. Notably, this significant improvement is related to the smaller *N*_th_(*E*), *J*_leak_, and *tanδ* of the PDMS/CCTO-15 wt% composite membrane compared with other tribo-dielectric materials. Figure 5G shows the variation trends of the *J*_out_, *V*_out_, and *P*_out_ of ECE-TENG at different resistances ranging from 10 to 10^9^ Ω. When *C*_ex_ and *f* are 22 nF and 3 Hz, respectively, the maximum instantaneous *P*_out_ reaches 6 W m^−2^ under an external load of 10^7^ Ω. The durability test result in Fig. 5H demonstrates an unobvious decrease in *σ*_out_ after 10,000 testing cycles, which indicates the excellent durability of the optimal ECE-TENG. The slight fluctuations in output performance may be due to long-term operation leading to surface wear of the friction layer. Table S2 compares the output performance of the organic–inorganic composite membrane-based TENG with those previously reported in the literature. The prepared ECE-TENG demonstrates promising output performance, indicating its potential for powering IoT micro-systems.

### Demonstration of the ILTRS

**Fig. 6.**
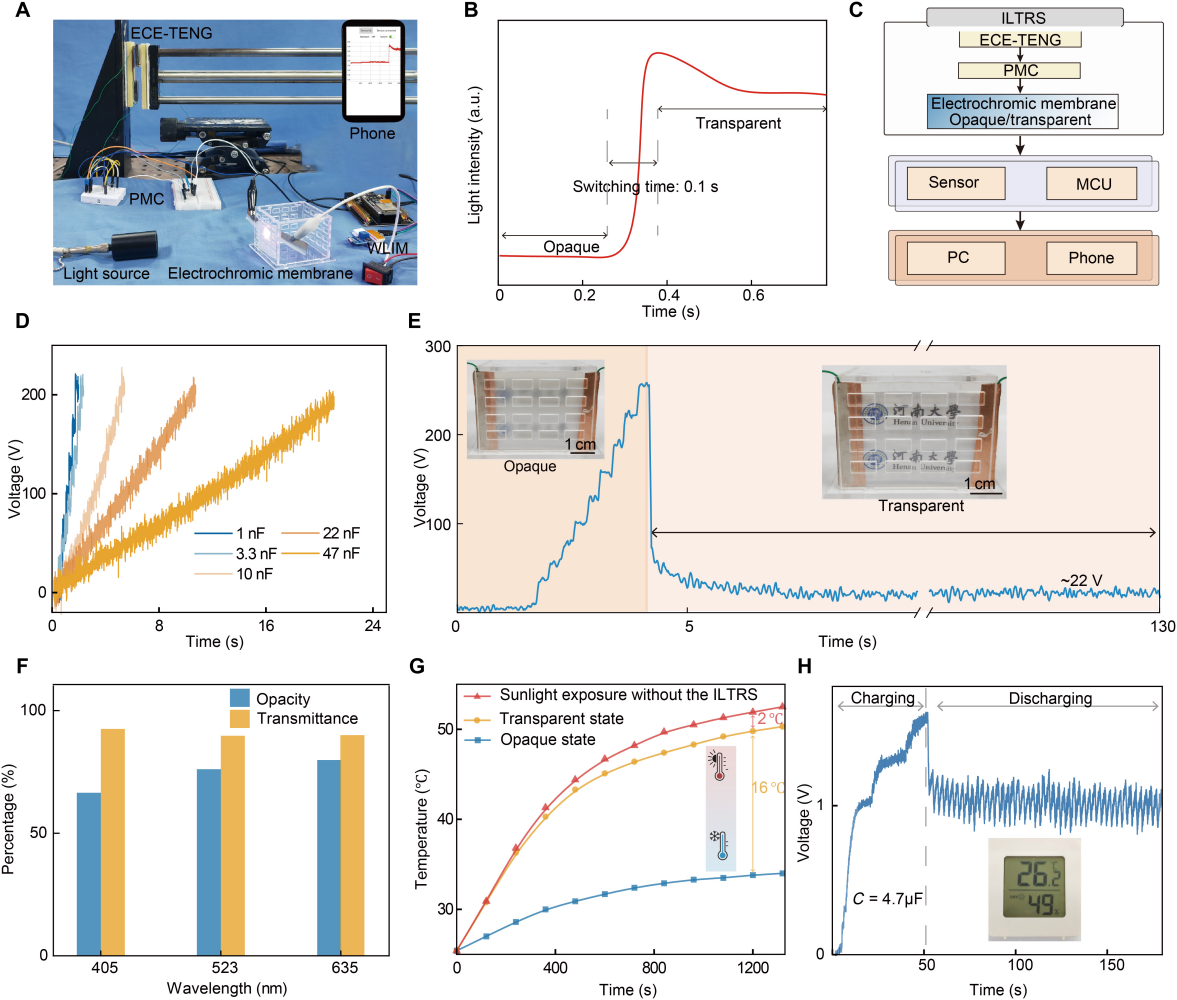
Demonstration of the indoor light-temperature regulation system (ILTRS). (A) Optical image of the ILTRS. (B) Light intensity feedback from its brightness sensor in different states. (C) Workflow diagram of the ILTRS. (D) Voltage curves of different capacitors charged by ECE-TENG. (E) Voltage curves during the process of charging and powering the electrochromic membrane. (F) Opacity and transmittance at different wavelengths for the ILTRS. (G) Dependence of the indoor temperature on time in the sunlight exposure without the ILTRS, transparent state, and opaque state. (H) Voltage curve during the process of charging and powering a thermohygrometer.

To verify the potential of ECE-TENG in the application of IoTs, an ILTRS consisting of PMC, ECE-TENG, and the electrochromic membrane was constructed for indoor light-temperature regulation. The optical image of the ILTRS and the brightness information received on the mobile phone are presented in Fig. 6A and B, respectively. The switching time of ILTRS is 0.1 s, indicating that ILTRS powered by ECE-TENG can switch quickly between the opaque and transparent states. As shown in the workflow diagram of the ILTRS in Fig. 6C, ECE-TENG converts the ambient mechanical energy into electrical energy. PMC rectifies and stores this energy and then powers the electrochromic membrane to switch between the opaque and transparent states, thus realizing the adjustment of indoor temperature and brightness. Subsequently, the brightness sensor sends the brightness information inside the building model wirelessly to the mobile phone, as demonstrated by Movie S1. Figure 6D presents the voltage curves of different capacitors charged by ECE-TENG, and the corresponding test circuit diagram is presented in Fig. S15. The results show that the charging time increases with the capacitance value. To quickly achieve the supply voltage of the electrochromic membrane, a 3.3-nF capacitor was selected to design PMC. As presented in Fig. 6E and Movie S2, ECE-TENG can provide a continuous and stable electric energy supply to the electrochromic membrane, achieving a switch between the transparency and the opaqueness of the electrochromic membrane. When the electrochromic membrane was continuously working (after 4.3 s in Fig. 6E), the voltage stabilized at approximately 22 V. To verify that a 22 V supply voltage can drive the electrochromic membrane, we used a programmable voltage source (GWINSTEK GPP-4323) to adjust the supply voltage while maintaining an output current of 0.05 mA, as shown in Fig. S16. It is noteworthy that the constant current of 0.05 mA exceeds the output current of TENG prepared in this work. Experimental results demonstrate that the electrochromic membrane functions normally within a supply voltage range of 5 to 25 V, confirming the feasibility of the 22 V supply voltage shown in Fig. 6E.

In addition, the opacity [40] and transmittance [41] of the electrochromic membrane were tested at light wavelengths of 405, 523, and 635 nm according to the formula in Note S4. The results in Fig. 6F indicate that the opacity and transmittance of the electrochromic membrane are higher than 62% and 87%, respectively. Figure 6G shows the dependence of the indoor temperature on time under illumination of AM 1.5 G in 3 different states. After 1,200 s, the difference in indoor temperature between the transparent and opaque states of the electrochromic membrane reaches 16 °C. The above experimental results show that ILTRS holds significant application prospects in saving energy used to regulate indoor light intensity and temperature. In addition, because of the excellent ECE-TENG output, it can easily and continuously drive the thermohygrometer shown in Fig. 6H. The detailed demonstration is shown in Movie S3.

## Conclusion

To sum up, the effects of trap state density, relative dielectric constant, leakage current density, and dielectric loss of the PDMS/CCTO composite membranes, and the work function difference between Cu electrode and composite membranes on the charge behaviors have been investigated, revealing the mechanism by which the TENG output is synergistically affected by charge generation, storage, and dissipation. The experimental results indicate that the output charge density of CS-TENG is positively affected by trap state density and relative dielectric constant of tribo-dielectric layers, and the work function difference between the electrode and tribo-dielectric layers, as well as negatively affected by leakage current density and dielectric loss. Although the CS-TENG output can be enhanced by optimizing the above factors, the maximum output charge density is only 17% of the theoretical value, which may be attributed to the insufficient contact electrification ability of PDMS/CCTO composite membranes to provide more triboelectric charges. Afterward, ECE-TENG was fabricated to get rid of the effect of contact electrification on the output charge density. ECE-TENG with PDMS/CCTO-15 wt% reaches the optimal balance among charge generation, storage, and dissipation, and thus, its output performance is superior to those of other samples. The highest output charge density of ECE-TENG was 236 μC m^−2^, which was 87% of the theoretical value, and the corresponding power density was 6 W m^−2^. Our core contribution lies not in a singular material or structural innovation, but rather in unveiling a universal synergistic mechanism within the TENG field through a new material system—a mechanism that is not dependent on specific materials. Although the PDMS/CCTO composite membrane was employed as the subject of this study, its role was to serve as an experimental platform for validating this proposed mechanism. Finally, an ILTRS based on ECE-TENG was proposed. The electrochromic membrane has an opacity and transmittance higher than 62% and 87% at wavelengths of 405, 523, and 635 nm, respectively, demonstrating an excellent ability to regulate light intensity. The difference in indoor temperature between the transparent and opaque states of the electrochromic membrane reaches 16 °C after 1,200 s. In summary, this work not only provides new insights for the development of high-performance triboelectric materials based on organic–inorganic composite membranes but also will accelerate the development of TENG toward large-scale IoT practical applications.

## Materials and Methods

### Preparation of CCTO and composition membranes

The PDMS main agent and curing agent were sourced from Shanghai Deji Trading Co. Ltd. The CCTO powder was purchased from Kramer Reagent Company. The preparation process of the PDMS/CCTO is presented in Fig. S1. Firstly, the curing agent was added dropwise to the PDMS main agent and stirred evenly (the mass ratio of the main agent PDMS to the curing agent is 10:1) to prepare the PDMS solution. Secondly, CCTO particles were mixed with PDMS solution and stirred at room temperature for 1 h. Notably, PDMS/CCTO composite membranes with different contents were synthesized by varying the CCTO contents to 0, 5, 10, 15, 20, and 25 wt%. Subsequently, the above mixture was spin-coated on glass (300 rpm/30 s to 2,000 rpm/5 s) in ambient conditions, followed by heating (80 °C, 2 h) to obtain PDMS/CCTO composite membranes with different CCTO contents. The commercial electrochromic membrane was cut to 4 cm × 5 cm for future employment.

### Preparation of ECE-TENG

ECE-TENG is composed of a main TENG, an external TENG, a half-wave rectifier bridge, and an external capacitor (*C*_ex_) in Fig. 1B. Among them, both the main TENG and an external TENG are composed of an acrylic plate (3 × 4 × 2 mm), foam (2 mm thick), silica gel layer (2 mm thick), Cu electrode, and PDMS/CCTO composite membranes. For the main TENG, the PDMS/CCTO composite membrane acted as a tribo-dielectric layer and was pasted to the Cu electrode (2 × 2.5 × 0.1 mm, chamfer is 5 mm), which is attached to the acrylic using foam tape. Another copper tape with the same area is used as both a friction layer and a counter electrode. For the external TENG, a Cu tape (3 × 2.5 × 0.1 mm chamfer is 5 mm) and FEP are used as friction materials. A half-wave rectifier and a capacitor form a charge excitation circuit linking the main and external TENGs.

### Measurement and characterization

The surface morphologies of samples were measured by a scanning electron microscope (Carl Zeiss Optics Co. Ltd., Gemini SEM 500). The XRD pattern was tested using an XRD instrument (Bruker D8 Advance, Cu Ka radiation). The thickness of all PDMS/CCTO composite membranes was 100 μm, which was measured using a micrometer screw gauge (Deqing Shengtaixin Electronic Technology Co. Ltd., Q2LF). The surface roughness and surface potential of the PDMS/CCTO composite membrane were measured by a scanning probe microscope (Oxford Instruments, MFP-3D Origin+). The *ε*_r_ and *tanδ* of the PDMS/CCTO composite membrane were measured by an impedance analyzer (Shanghai Yanghe Electronic Technology Co. Ltd., TZDM-RT-1000). The leakage current density is measured by a high-voltage amplifier (Trek model 10/10B) and a high-resistance meter (Keithley 6517B). Ionized injection for the introduction of the surface charges was achieved by an ion gun (UK Milty, Zerostat 3). The surface potential attenuation results of the PDMS/CCTO composite membrane were measured by a high-speed electrostatic voltmeter (Trek 370). TENG was operated by a linear motor (LinMot, P01-37). An electrostatic meter (Keithley 6514) was used to measure the electrical TENG output. Different wavelength light sources are composed of lasers (Ningbo Yuanxin Optoelectronic Technology Co. Ltd., 405/250 mW, 532/400 mW, 635/400 mW). The 1 mW cm^−2^ solar illumination was provided by a simulated daylight xenon lamp light source system (Zhongjiao Jinyuan, CEL-S500).

## Data Availability

Data are available from the corresponding author upon reasonable request.

## Supplementary Materials

Supplementary 1 Figs. S1 to S15 Notes S1 to S4 Tables S1 and S2 Movies S1 to S3 References [42–50]
