# Supplementary material for: Achieving 87% of Theoretical Output Charge Density by Optimizing Charge Behaviors in Polydimethylsiloxane/CaCu3Ti4O12-Based Triboelectric Nanogenerators
Source: Research (Wash D C). 2025 Oct 9;8:0921. doi: 10.34133/research.0921 (PMC12508525; doi:10.34133/research.0921)
Supplement: Supplementary 1 — Figs. S1 to S15 Notes S1 to S4 Tables S1 and S2 Movies S1 to S3 References [42–50] [file research.0921.f1.zip › Supplementary Material.docx]

**Supporting Information**

**Achieving 87% of Theoretical Output Charge Density by Optimizing Charge Behaviors in Polydimethylsiloxane/CaCu_3_Ti_4_O_12_-based Triboelectric Nanogenerators**

Jinyang Liu^1^, Zhongkun Wang^1^, Shuo Wang^1^, Yuanzheng Zhang^1*^, Weikun Li^1^, Song Zhao^1^, Dongyang Li^1^, Yonghui Wu^1^, Hengyu Guo^2*^, Haiwu Zheng^1*^

^1^Henan Key Laboratory of High Efficiency Energy Conversion Science and Technology, Henan Province Engineering Research Center of Smart Micro-nano Sensing Technology and Application, School of Physics and Electronics, Henan University, Kaifeng 475004, PR China

^2^School of Physics, Chongqing University, Chongqing, 400044, China

* Address correspondence to: zhangyz0926@henu.edu.cn (Y. Z. Zhang); [physghy@cqu.edu.cn](mailto:physghy@cqu.edu.cn) (H. Y. Guo); zhenghaiw@ustc.edu (H. W. Zheng).

**Table of Contents**

**Supplementary Figures S1-S15**…………………………………………..….…...**3-17**

**Supplementary Notes S1-S4**………………………………………..…………....**18-20**

**Supplementary Tables S1-S2**…………………………………………………..……**21**

**Supplementary Movies S1-S3**….………………………………………………..….**22**


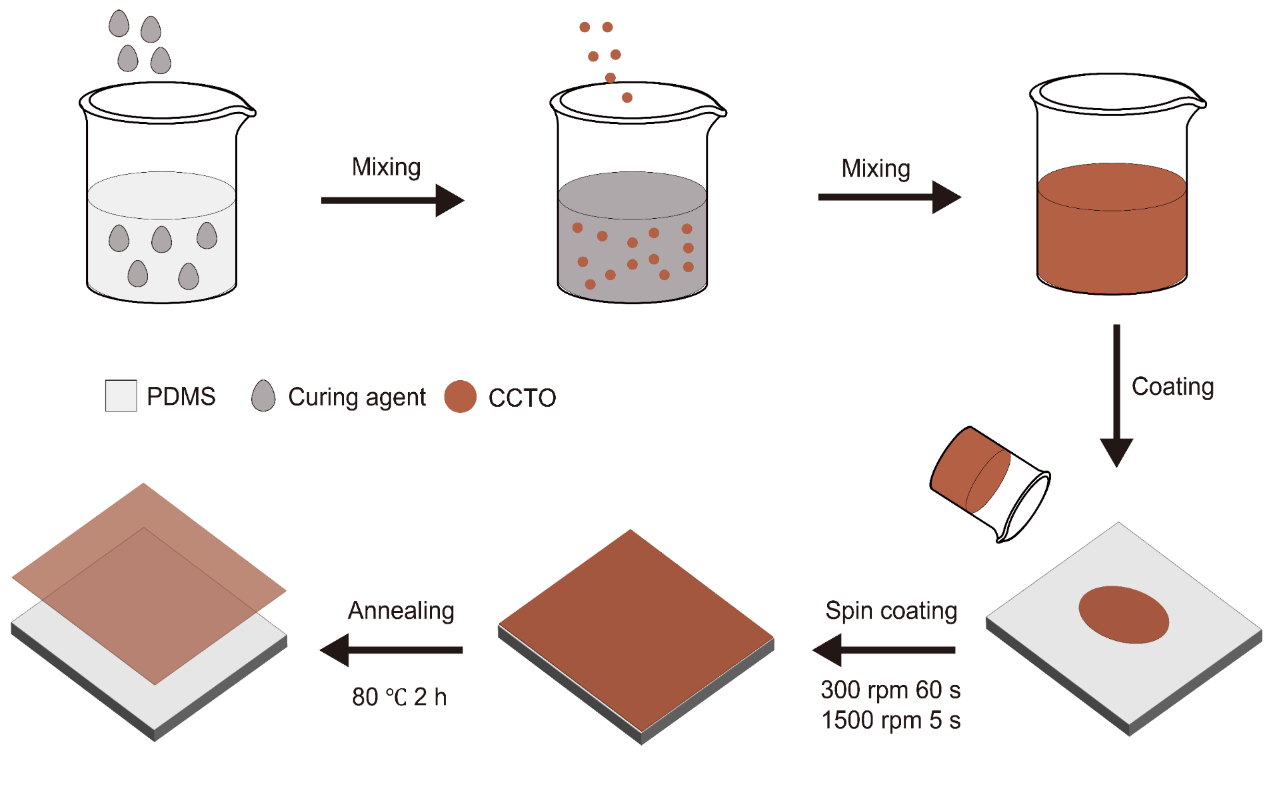


**Figure S1.** The manufacturing process of PDMS/CCTO composite membranes.


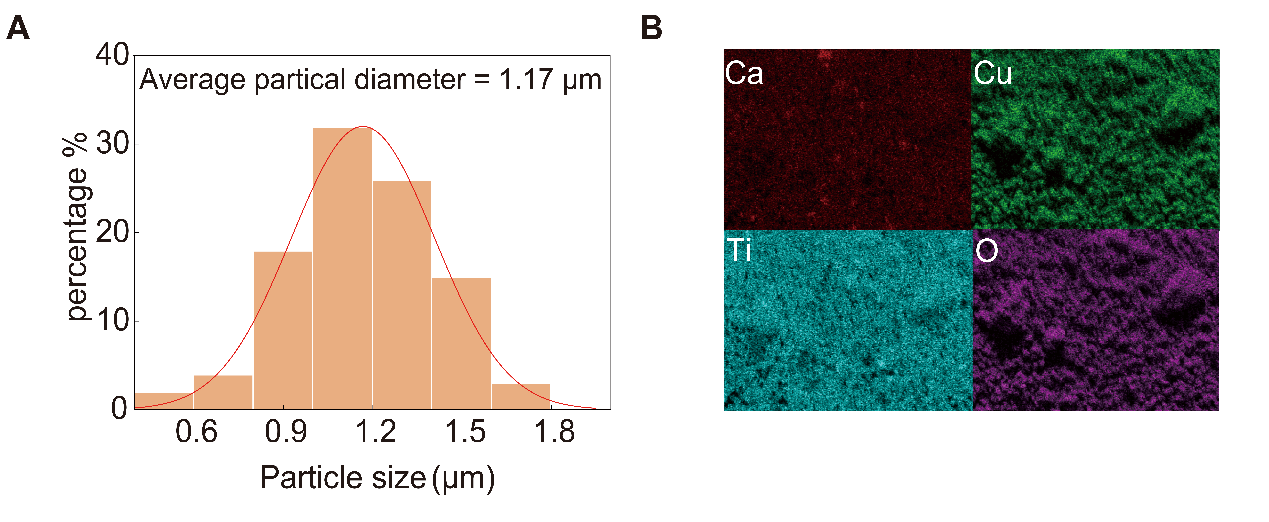


**Figure S2.** (a) Average particle diameter of CCTO particles. (b) EDX map of CCTO particles.


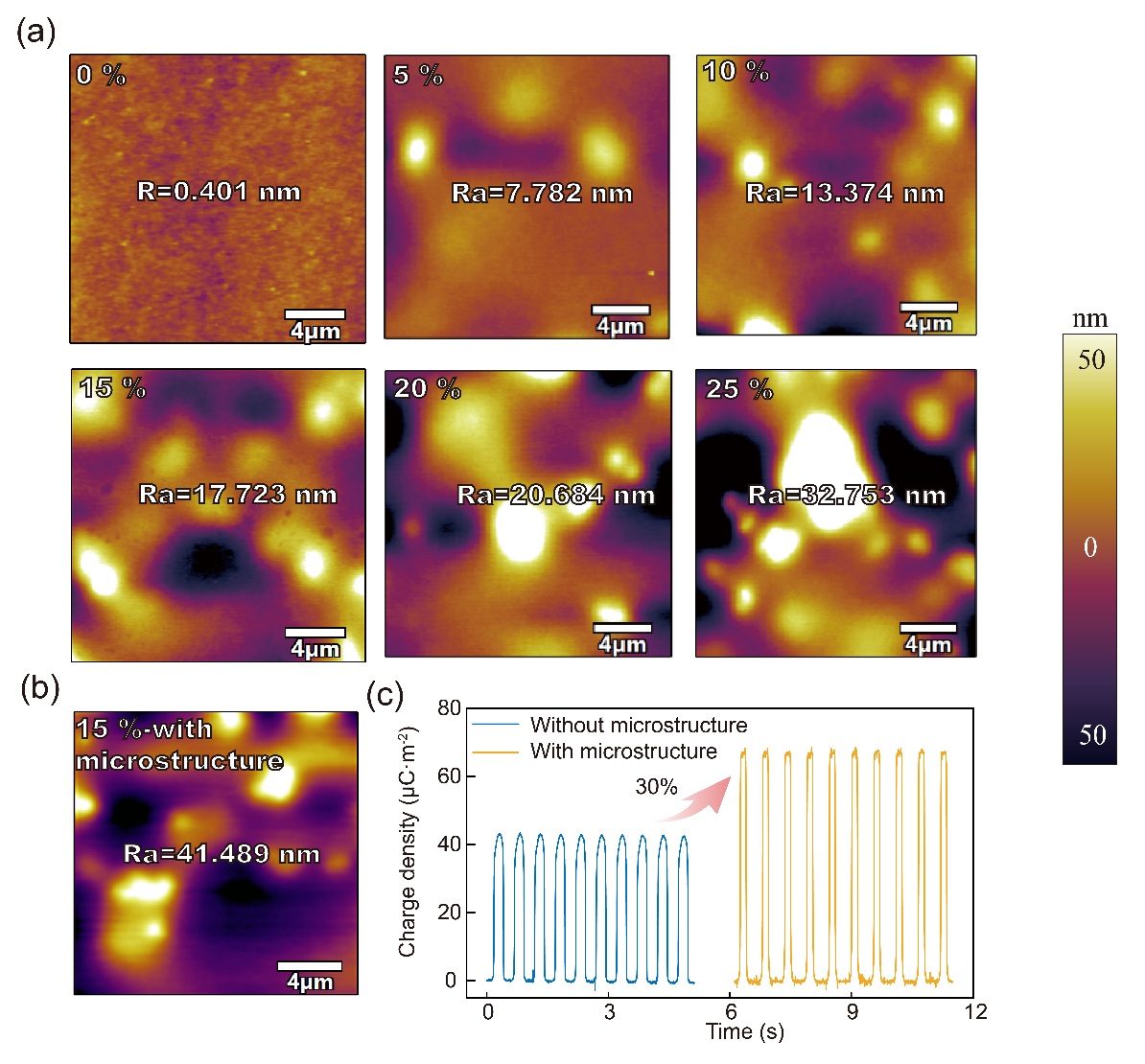


**Figure S3.** (a) AFM results of composite membranes with different CCTO contents. (b) AFM results of PDMS/CCTO-15 wt% composite membrane with surface microstructure. (c) Comparison of *σ*_out_ with and without microstructured PDMS/CCTO-15 wt% composite membrane as tribo-dielectric layer.


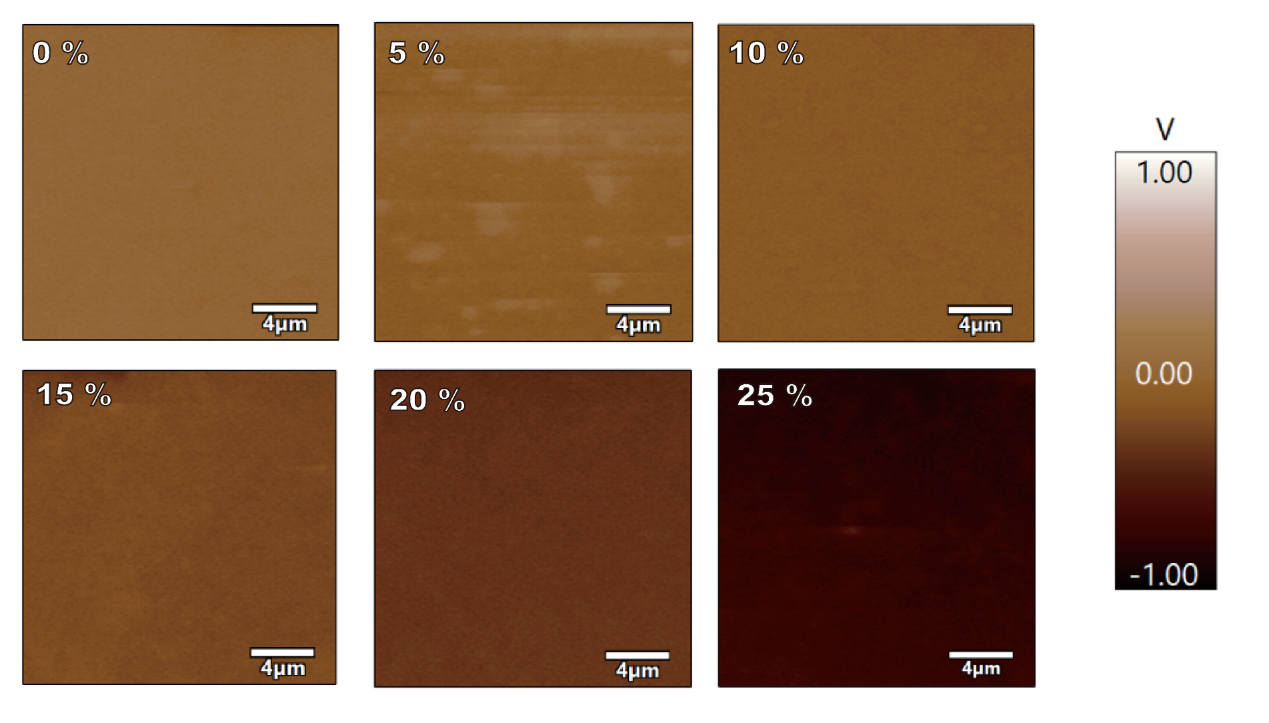


**Figure S4.** Contact potential difference of composite membranes with different CCTO contents.


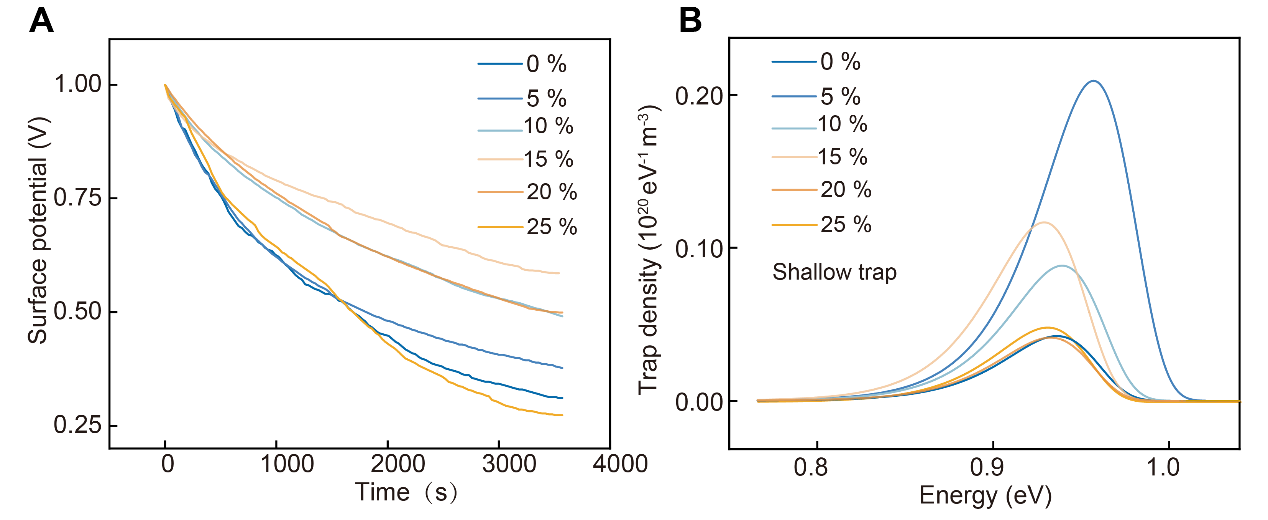


**Figure S5.** (a) Surface potential decay of the electron capture capability of the composites after being charged. (b) Electron shallow trap state density versus trap state energy calculated by the ISPD model.


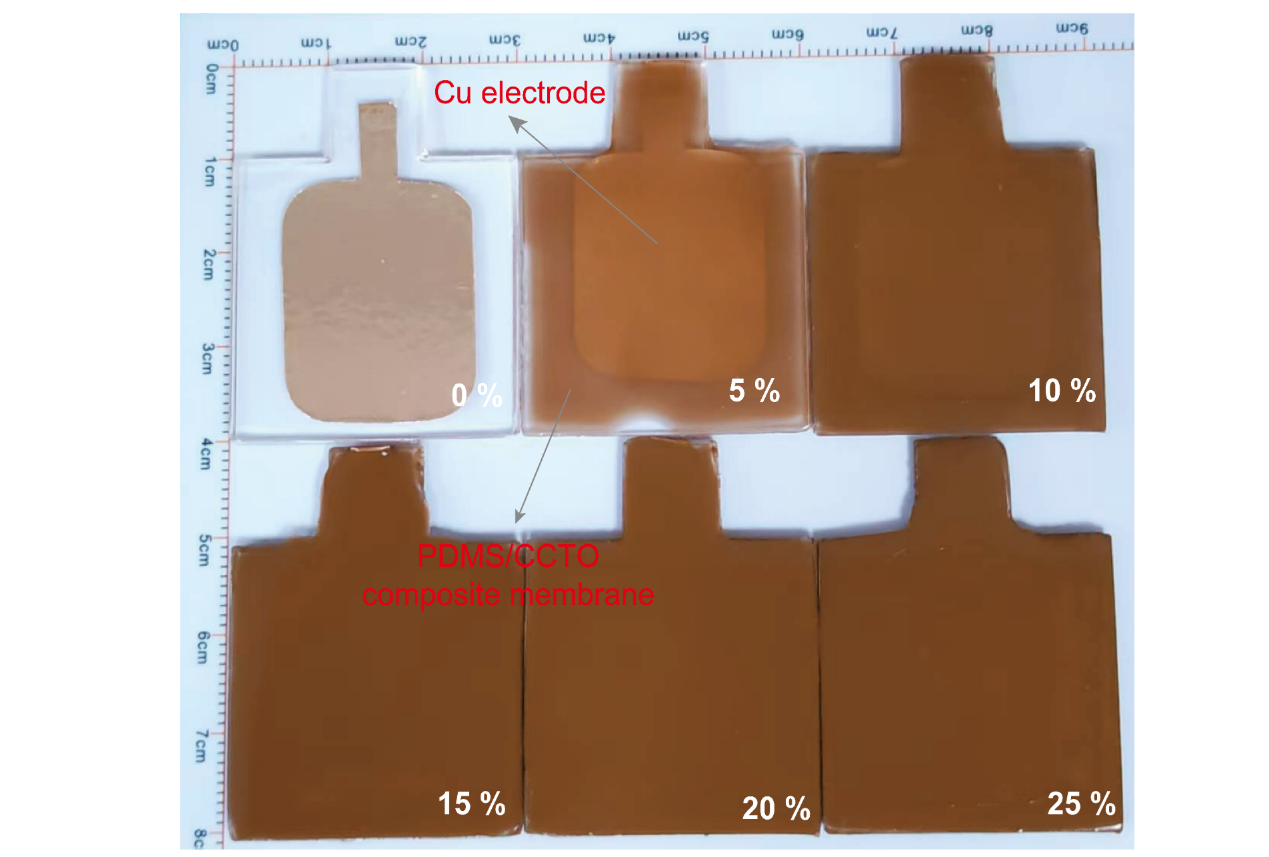


**Figure S6.** Optical images of PDMS/CCTO composite membranes with different contents and their corresponding bottom electrodes.


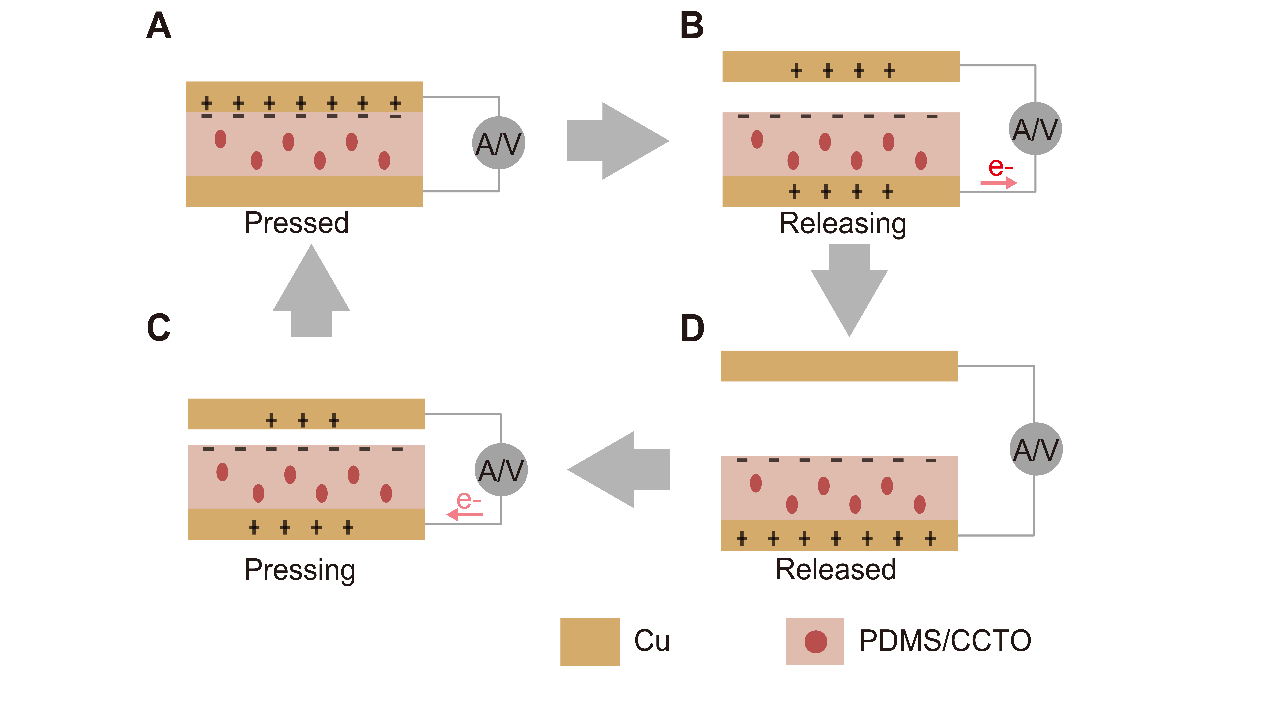


**Figure S7.** Working principle of the PDMC/CCTO based on the CS-TENG.

As shown in Figure S5, when the tribo-dielectric layer of CS-TENG comes into contact, charge transfer occurs due to the difference in their work functions, and the Cu and PDMS/CCTO composite membranes are positively and negatively charged, respectively. When the tribo-dielectric separates, the triboelectric charge will generate a time-varying electric field in space, which will drive free electrons to transfer directionally from the top electrode to the bottom electrode in the external circuit until electrostatic equilibrium is reached. Similarly, when the two tribo-dielectric return to their initial contact state, electrons will flow in reverse to the top electrode until they are completely in contact. At this point, the device has completed one cycle of operation and generated an alternating electrical signal.


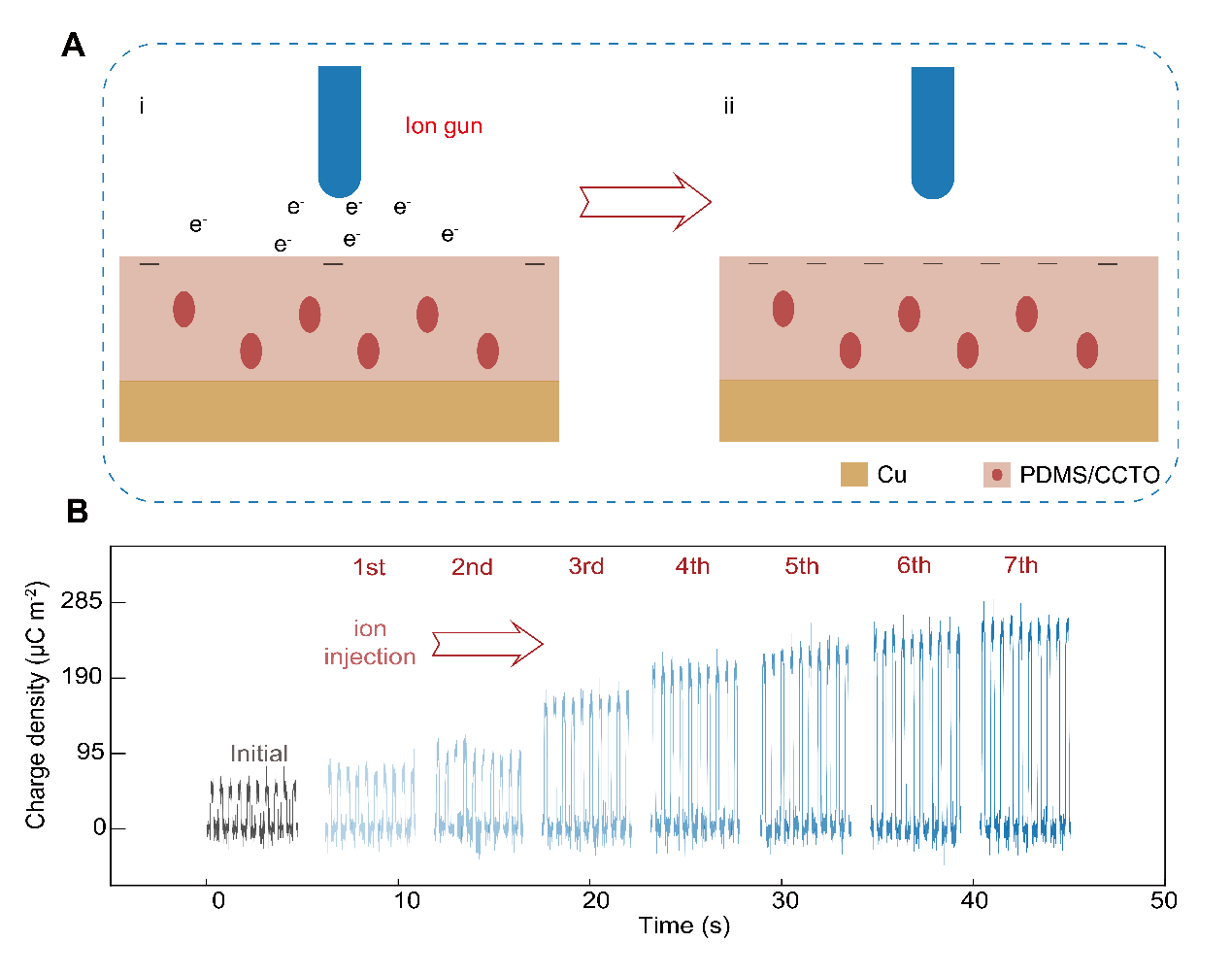
**Figure S8.** (a) Structural schematic of the ion injection charging. (b) Relationship between the number of injections and the output charge density (*σ*_out_).


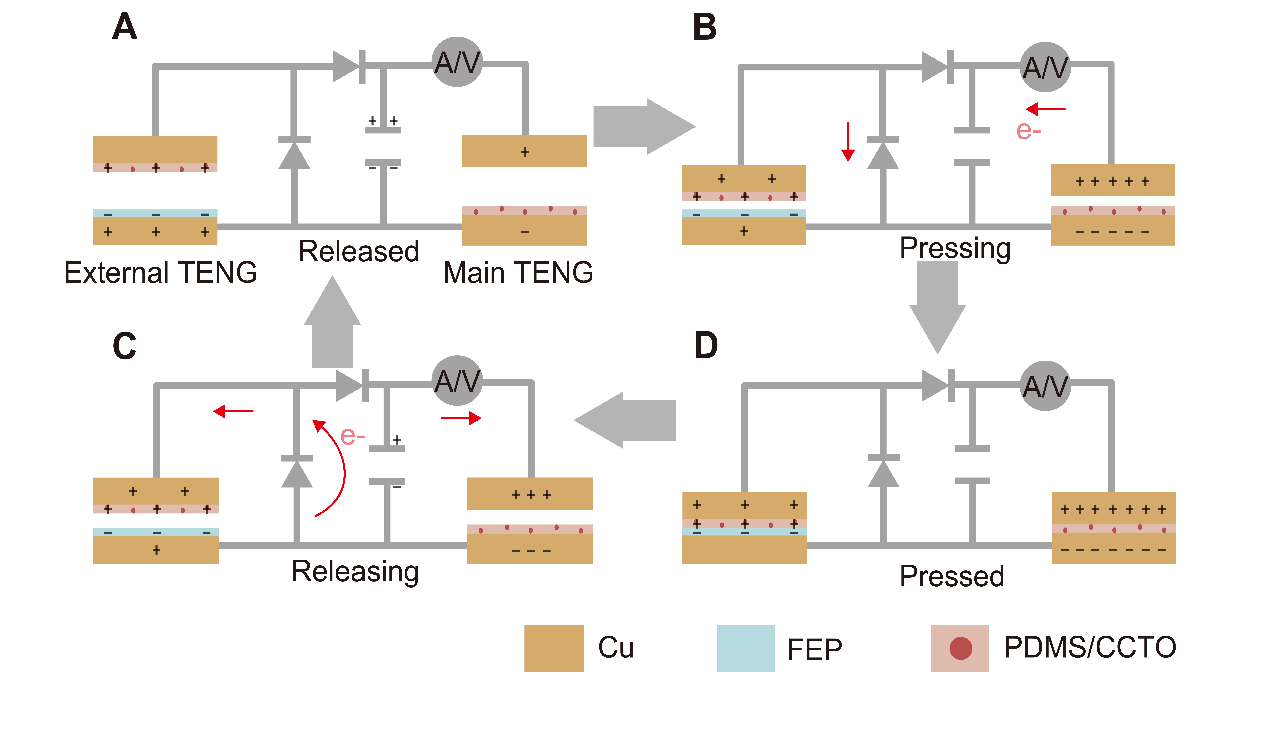


**Figure S9.** Working principle of the PDMC/CCTO based on the ECE-TENG.


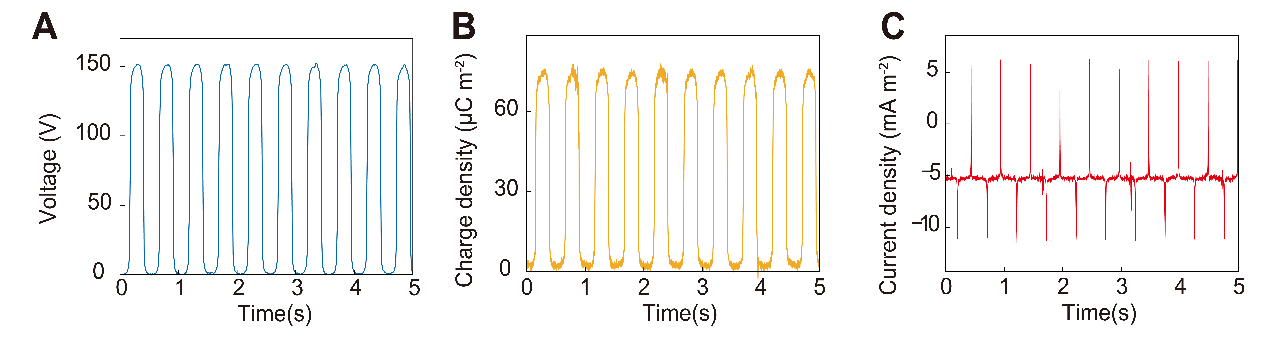


**Figure S10.** (a) Open voltage of excitation TENG. (b) Charge density of excitation TENG. (c) Current density of excitation TENG.


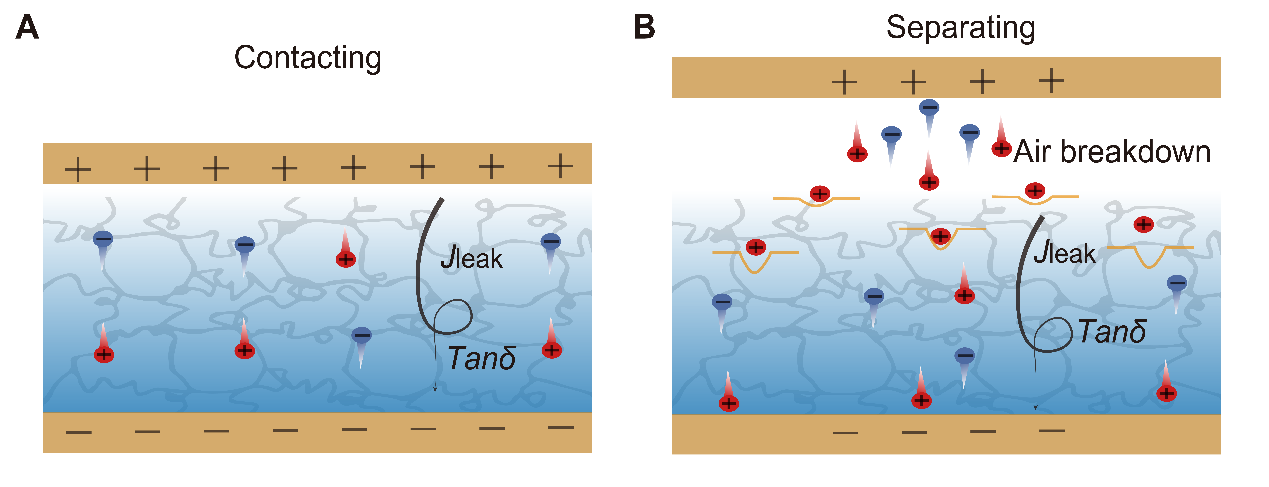


**Figure S11.** Schematic diagram of the effect of air breakdown, leakage current density, and dielectric loss on output suppression in the main TENG.

Charge dissipation can be divided into two aspects [22]. Charge deposition on the surface of the tribo-dielectric layer and charge loss inside, as shown in Figure S11. On the one hand, weak bound carriers and dipoles inside the material move in one direction under an electric field, leading to the accumulation of positive charges on the bottom electrode surface and negative charges on the top electrode, resulting in a decrease in the amount of charge transferred from the main TENG to the external capacitor during the separation process. On the other hand, the directional electric field generated by high charge density causes air ionization and charge injection into the dielectric surface or inside, resulting in *σ*_out_ loss during the separation process. It is worth noting that this is closely related to the trap state density, leakage current density, and dielectric loss of the tribo-dielectric layer.


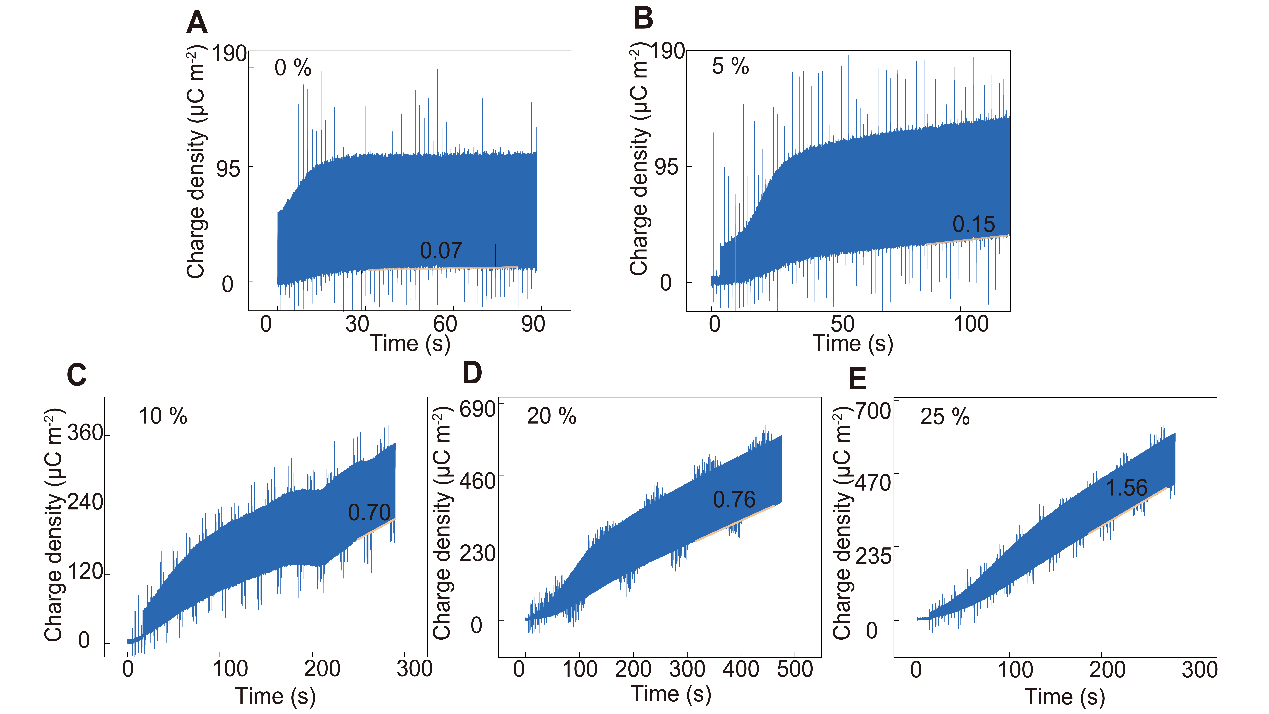


**Figure S12.** (a-e) Charge density curve for charge excitation of PDMS/CCTO at composite contents of 0、5、10、20、25 wt%.


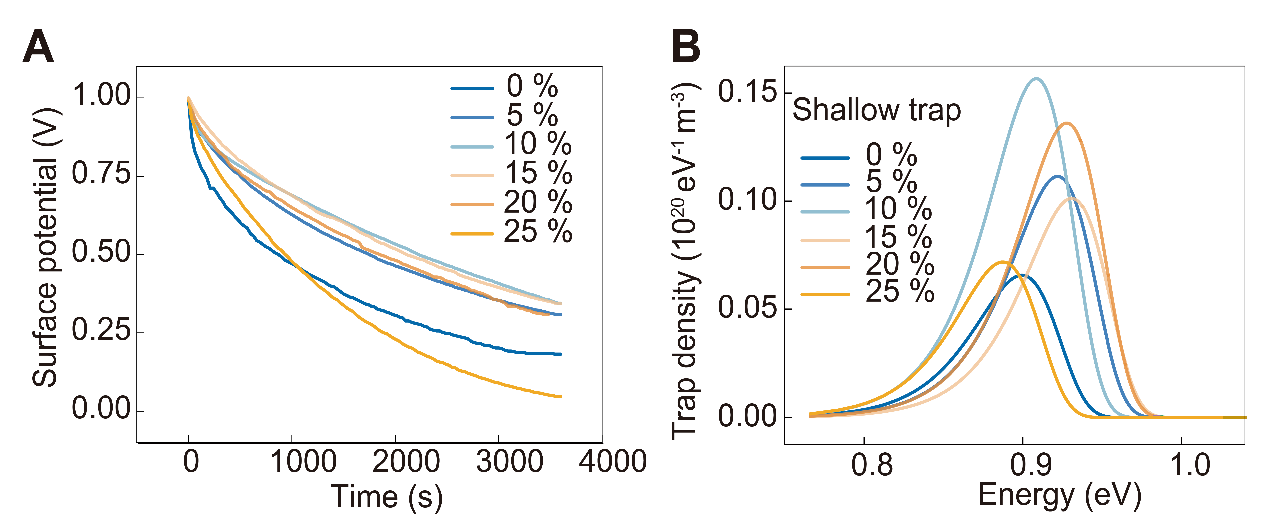


**Figure S13.** (a) Surface potential decay of hole capture capability of the PDMS/CCTO composite membranes with different CCTO contents after being charged. (b) Hole shallow trap state density versus trap state energy calculated by the ISPD model.


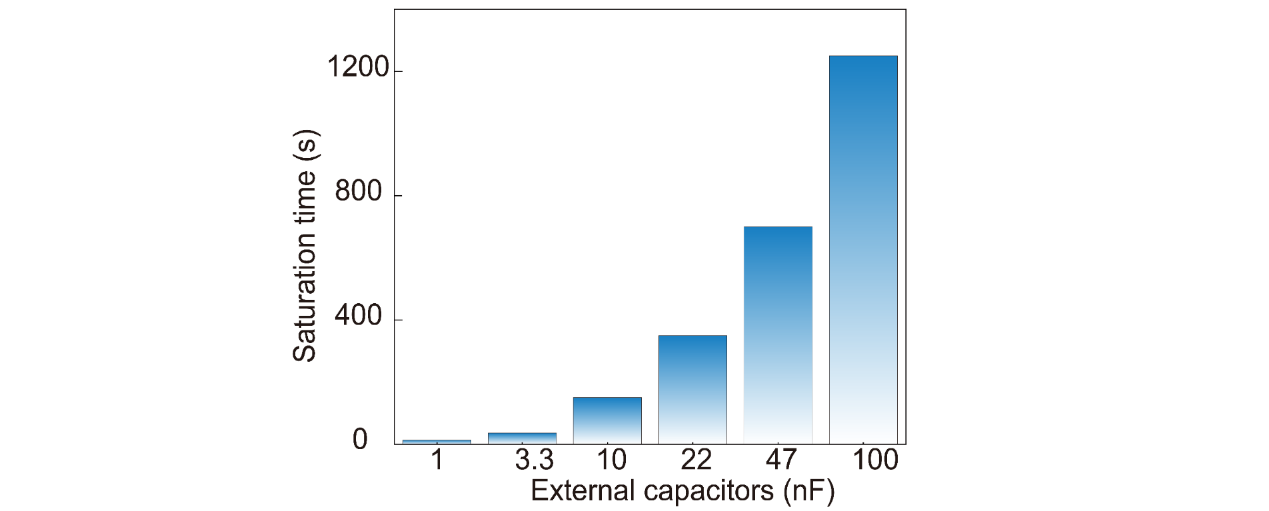


**Figure S14.** Output saturation time of the ECE-TENG under different external capacitors.


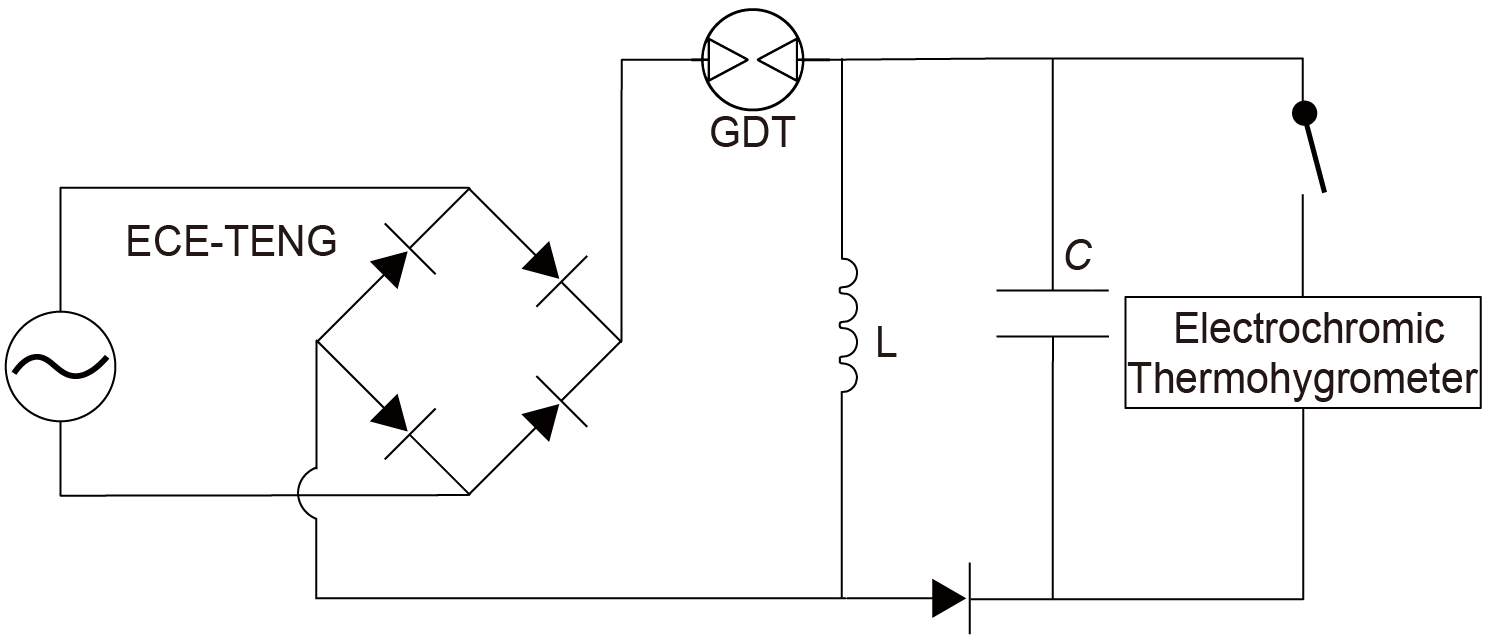


**Figure S15.** Power management circuit for the electrochromic membrane and thermohygrometer.


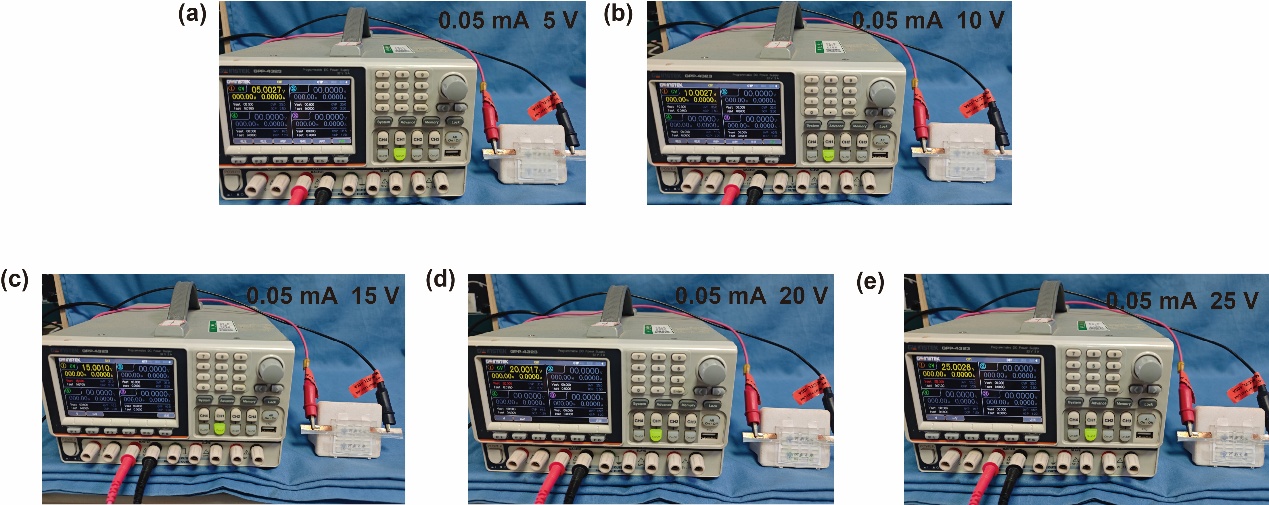


Fig.16 (a-e) Electrochromic membrane powered by different supply voltage under an output current of 0.05 mA

**Note S1.** The effective work function of the PDMS/CCTO composite membranes.

The actual measured value *CPD* of the KPFM is expressed as [42]:

$\begin{matrix} CPD=\emptyset_{sample}-\emptyset_{probe}=\frac{W_{sample}-W_{probe}}{-e} \end{matrix}$ (S1)

Where *Ф* is _S_urface potential, *W* is _W_ork function, and *e* is the unit charge (e = 1.6×10^-19^ C).

Derived from Equation (1), the sample work function is expressed as:

$\begin{matrix} W_{sample}=-e\cdot CPD+W_{probe} \end{matrix}$ (S2)

**Note S2.** Calculate the trap state energy level and the corresponding trap state density.

Considering that there are two types of decay processes occurring at the same time, to further obtain the attenuation curve of the tribo-dielectric layer surface potential, the double exponential attenuation equation can be applied to fit the attenuation curve [43]:

$\begin{matrix} V\left(t\right)=Aexp^{-\frac{t}{B}}+Cexp^{-\frac{t}{D}} \end{matrix}$ (S3)

Where *A* and *B* are the shallow trap parameters. *C* and *D* are the deep trap parameters. *t* is the time.

The trap state density *N*(*E_T_*) of a dielectric material is expressed as [21]:

$\begin{matrix} N\left(E_{T}\right)=\frac{\varepsilon_{0}\varepsilon_{r}}{ef_{0}K_{B}TL\delta}\left|t\frac{dV\left(t\right)}{dt}\right| \end{matrix}$ (S4)

Where *ε_0_* and *ε_r_* are the vacuum permittivity and the relative permittivity of the tribo-dielectric layer. The *e* is the unit charge(1.602×10^-19^C). *f*_0_ is the initial occupancy of the hole and electron (set *f*_0_ = 1). *K*_B_ is the Boltzmann constant (1.38×10^-23^ J K^-1^). *T* is the room temperature (set to 300 K). *L* is the thickness of the tribo-dielectric layer. *δ* is the range of an even charge distribution from the surface (*set δ = L*).

Derived from Equation(1)(3), The shallow trap state density *N*_shallow_(*E_T_*) and the deep trap state density *N*_deep_(*E_T_*) are expressed as：

$$\begin{matrix} N_{shallow}\left(E_{T}\right)=\frac{\varepsilon_{0}\varepsilon_{r}}{ef_{0}K_{B}TL\delta}\frac{A}{B}exp^{-\frac{t}{B}} \end{matrix}$$

$\begin{matrix} N_{deep}\left(E_{T}\right)=\frac{\varepsilon_{0}\varepsilon_{r}}{ef_{0}K_{B}TL\delta}\frac{C}{D}exp^{-\frac{t}{D}} \end{matrix}$ (S5)

The trap state energy *E*_T_ of a dielectric material is expressed as$:$

$E_{T}=K_{B}Tln\left(\gamma t\right)$ (S6)

where, 𝛾 is the attempt-to-escape frequency of the trapped electrons (set 𝛾 = 2×10^13^s^-1^).

**Note S3.** Theoretical calculation of the maximum surface charge density limited by air breakdown in TENG.

ECE-TENG can realize the gradual accumulation of charge on the main TENG electrode. When the accumulated charge density exceeds a certain value, the gap voltage exceeds the air breakdown voltage, resulting in air breakdown. In a word, the air breakdown limits the maximum value of ECE-TENG [36].


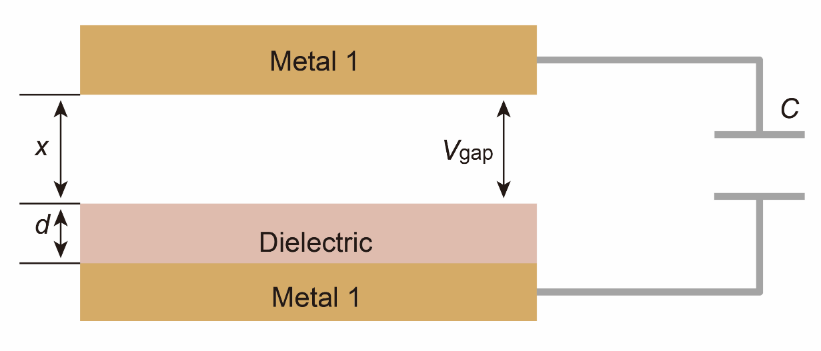


During the contact-separation process, the voltage of the TENG is always equal to the voltage of the external capacitor, denoted as:

$\begin{matrix} V_{TENG}\left(x\right)=V_{C}\left(x\right) \end{matrix}$ (S7)

The capacitance of TENG is:

$\begin{matrix} C_{TENG}\left(x\right)=\frac{Q\left(x\right)}{V_{TENG}\left(x\right)}=\frac{\varepsilon_{0}S}{\frac{d}{\varepsilon_{r}}+x} \end{matrix}$ (S8)

*ε*_0_ and *ε*_r_ are the vacuum permittivity and the relative permittivity of the dielectric layer, respectively. *d*, *S* and *x* are the dielectric layer thickness, area and air gap distance, respectively. *Q*(*x*) is the amount of charge of TENG when the air gap is *x*.

The external capacitance is:

$\begin{matrix} C=\frac{Q_{C}+\left(Q_{0}-Q\left(x\right)\right)}{V_{C}\left(x\right)} \end{matrix}$ (S9)

The *Q*_C_ *Q*_0_ assumes the charge of the external capacitor and the TENG when the main TENG is in contact, respectively.

Derived from Equation (1)(2)(3):

$\begin{matrix} \frac{Q\left(x\right)}{C_{TENG}\left(x\right)}=\frac{Q_{C}+\left(Q_{0}-Q\left(x\right)\right)}{C} \end{matrix}$ (S10)

When x=0：

$\begin{matrix} \frac{Q\left(0\right)}{C_{TENG}\left(0\right)}=\frac{Q_{C}}{C} \end{matrix}$ (S11)

Derived from Equation (2)(4)(5), the *σ*_max_ of TENG at an air gap of *x*：

$\begin{matrix} \sigma\left(x\right)=\frac{Q\left(x\right)}{S}=\sigma_{0}\left(1-\frac{x}{\frac{d}{\varepsilon_{r}}+x+\frac{\varepsilon_{0}S}{C}}\right) \end{matrix}$ (S12)

Then, the gap voltage between dielectric and metal 1 can be expressed as:

$\begin{matrix} V_{gap}\left(x\right)=\frac{x\sigma_{0}}{\varepsilon_{0}}\left(1-\frac{x}{\frac{d}{\varepsilon_{r}}+x+\frac{\varepsilon_{0}S}{C}}\right) \end{matrix}$ (S13)

According to Paschen's law, the air brekdown voltage can be expressed as follows [15]:

$\begin{matrix} V_{B}=\frac{A\left(Px\right)}{\ln\left(Px\right)+B} \end{matrix}$ (S14)

Where *P* is the atmospheric pressure (*P*=1.01×10^5^ Pa) . 𝐴 and 𝐵 are constants determined by the air composition and pressure (*A*=271.4，*B*=1.08).

$\begin{matrix} V_{gap}\left(x\right)\leq V_{B} \end{matrix}$ (S15)

Derived from Equation (7)(8)(9), The maximum charge density *σ*_max_ is：

$\begin{matrix} \sigma_{max}=\left[\frac{AP\varepsilon_{0}}{\left(\ln(Px\right)+B)\left(1-\frac{x}{\frac{d}{\varepsilon_{r}}+x+\frac{\varepsilon_{0}S}{C}}\right)}\right]_{min} \end{matrix}$ (S16)

In the short-circuit state, the theoretical maximum charge density *σ*_max_ is [44]:

$\begin{matrix} \sigma_{max}=\left[\frac{AP(\varepsilon_{0}+\frac{\varepsilon_{0}\varepsilon_{r}}{d}x)}{\left(\ln(Px\right)+B)}\right]_{min} \end{matrix}$ (S17)

**Note S4.** The calculation formula for transmittance and opacity of the electrochromic membrane.

The actual measured value, Transmittance (*T*) of the electrochromic membrane, is expressed as [41]:

$\begin{matrix} T=\frac{(I_{2}-I_{3})}{\left(I_{4}-I_{3}\right)}\times100\% \end{matrix}$ (S18)

The actual measured value, Opacity (*O*) of the electrochromic membrane, is expressed as [40]:

$\begin{matrix} O=\frac{(I_{4}-I_{3})-\left(I_{1}-I_{3}\right)}{\left(I_{4}-I_{3}\right)}\times100\% \end{matrix}$ (S19)

Where *I*_1_ is the light intensity of the opaque electrochromic membrane illuminated by the light source; *I*_2_ is the light intensity of the transparent electrochromic membrane when illuminated by the light source; *I*_3_ is the ambient light intensity; The intensity of light when the *I*_4_ light source is directly exposed.

**Table S1.** Comparison of experimental and theoretical values of *σ*_out_ of charge excited TENGs.

| Tribo-layer  Materials | *J*_leak_ | *tanδ* | *N*_t_(*E*)  (eV^-1^ m^-3^) | σ_test_/  σ_theory_ | Ref |
| --- | --- | --- | --- | --- | --- |
| PVDF | 2.8 μA@500 V | \ | >10^22^ | 15% | ^[45]^ |
| P(VDF-TrFE-CFE) | >10^-8^ A cm^-2^  @300 V | \ | \ | 42% | ^[20]^ |
| PVDF-PI | >10 pA@300 V | \ | \ | 74% | ^[22]^ |
| PDMS/CCTO | <10^-10^ A cm^-2^  @300 V | <0.015 | <10^20^ | 87% | This work |

**Table S2.** Comparison of the output performance of organic inorganic composite membranes as tribo-dielectric layers.

| Tribo-layer  Materials | Charge  Density  (μC m^−2^) | Current Density (mA m^-2^) | Output Voltage  (V) | Power Density  (W m^-2^) | Ref |
| --- | --- | --- | --- | --- | --- |
| BMF/CCTO | \ | 25.8 | 268 | \ | ^[33]^ |
| PDMS/TiO_2_ | 10 | 0.8 | 125 | 1 | ^[46]^ |
| PDMS/BaTiO_3_ | 53 | 2 | 72.2 | 0.14 | ^[47]^ |
| MPU/CCTO | 80 | 11.5 | 100 | 3.3 | ^[48]^ |
| PDMS/CCTO@BT | 50 | 22.1 | 291.14 | 1.34 | ^[34]^ |
| MSF/CCTO | 65 | 8 | 85 | 2.8 | ^[49]^ |
| PDMS/CCTO | 70 | 16.25 | 250 | 3.15 | ^[50]^ |
| PDMS/CCTO | 236 | 60 | 264 | 6.03 | This work |

**Movie S1.** The electrochromic membrane is powered by the ECE-TENG with PDMS/CCTO-15 wt% composite membrane.

**Movie S2.** Continuously power the electrochromic membrane via the ECE-TENG with PDMS/CCTO-15 wt% composite membrane.

**Movie S3.** Continuously power the thermohygrometer via the ECE-TENG with PDMS/CCTO-15 wt% composite membrane.
